# Huntingtin associates with the actin cytoskeleton and α-actinin isoforms to influence stimulus dependent morphology changes

**DOI:** 10.1371/journal.pone.0212337

**Published:** 2019-02-15

**Authors:** Adelaide Tousley, Maria Iuliano, Elizabeth Weisman, Ellen Sapp, Heather Richardson, Petr Vodicka, Jonathan Alexander, Neil Aronin, Marian DiFiglia, Kimberly B. Kegel-Gleason

**Affiliations:** 1 Laboratory of Cellular Neurobiology, Department of Neurology, Massachusetts General Hospital, Charlestown, Massachusetts, United States of America; 2 Department of Medicine and Cell Biology, University of Massachusetts Medical School, Worcester, Massachusetts, United States of America; Grenoble Institut des Neurosciences, FRANCE

## Abstract

One response of cells to growth factor stimulus involves changes in morphology driven by the actin cytoskeleton and actin associated proteins which regulate functions such as cell adhesion, motility and in neurons, synaptic plasticity. Previous studies suggest that Huntingtin may be involved in regulating morphology however, there has been limited evidence linking endogenous Huntingtin localization or function with cytoplasmic actin in cells. We found that depletion of Huntingtin in human fibroblasts reduced adhesion and altered morphology and these phenotypes were made worse with growth factor stimulation, whereas the presence of the Huntington’s Disease mutation inhibited growth factor induced changes in morphology and increased numbers of vinculin-positive focal adhesions. Huntingtin immunoreactivity localized to actin stress fibers, vinculin-positive adhesion contacts and membrane ruffles in fibroblasts. Interactome data from others has shown that Huntingtin can associate with α-actinin isoforms which bind actin filaments. Mapping studies using a cDNA encoding α-actinin-2 showed that it interacts within Huntingtin aa 399–969. Double-label immunofluorescence showed Huntingtin and α-actinin-1 co-localized to stress fibers, membrane ruffles and lamellar protrusions in fibroblasts. Proximity ligation assays confirmed a close molecular interaction between Huntingtin and α-actinin-1 in human fibroblasts and neurons. Huntingtin silencing with siRNA in fibroblasts blocked the recruitment of α-actinin-1 to membrane foci. These studies support the idea that Huntingtin is involved in regulating adhesion and actin dependent functions including those involving α-actinin.

## Introduction

Cellular spreading and morphology changes in response to stimulation are dependent on the function of actin and its associated proteins, and involve restructuring of focal adhesions [1–4]. Huntingtin is the protein product of HTT, a gene which is mutated in Huntington’s disease (HD) when a normal CAG repeat is expanded to >37 [5] and has been implicated in diverse cellular functions including vesicle trafficking and signaling functions at membranes (reviews [6, 7]). Huntingtin is an essential protein that is present in all cells, enriched in neurons, and is required for embryonic survival and survival of mature neurons in vivo [8–12]. Studies have suggested that Huntingtin interacts with molecular complexes that regulate actin dependent functions and that mutant Huntingtin alters these effects: In PC12 cells, polyglutamine expansion in exogenously expressed Huntingtin disrupted its association with the receptor tyrosine kinase TrkA, caused constitutive phosphorylation of TrkA receptor, and inhibited neurite outgrowth in response to stimulus [13]. Mutant Huntingtin exon1 toxicity can be modulated through ROCK inhibition and profilin phosphorylation [14]. Large scale protein interaction assays based on immunoprecipitations and mass spectrometry (MS) and yeast two-hybrid (Y2H) experiments and bioinformatics analyses also suggest that Huntingtin associates with proteins involved with the actin cytoskeleton including actin itself [15–17]. Of note, a purified fragment of Huntingtin was shown to directly interact with actin *in vitro* through its first 14 amino acids [18]. Endogenous Huntingtin co-localized to and is necessary for stress-induced nuclear actin/cofilin rods which are also altered in HD cells [19]. However, there has been limited evidence linking endogenous Huntingtin localization or function with cytoplasmic actin in cells [17, 20].

Growth factor stimulation and PI 3-kinase signaling in fibroblasts and other cells has been used as a classic model for examining the role of actin and its associated proteins in modulating cell morphology [21, 22]. In normal growth medium containing serum, fibroblasts take on numerous morphologies and are in various states of adhesion. Prior to stimulation, cells are grown in the absence of serum which contains numerous growth factors or other small molecules that activate signaling pathways. Cell maintenance in serum-free conditions causes many cells to senesce, attenuate numerous signaling pathways, and establish stabile cell-matrix contacts and large bundles of filamentous actin (F-actin) that traverse the cell known as stress fibers. Receptor tyrosine kinase stimulation of serum-starved primary fibroblasts with PDGF initiates PI 3-kinase activity, a transient, local rise of PI(3,4)P2 and PI(3,4,5)P3, recruitment of protein complexes and actin remodeling which can be visualized by labeling F-actin. This response involves formation of local protrusions, lamellipodia (thin cytoplasmic membrane extensions from the leading edge of cells that are rich in F-actin, devoid of organelles and contain adhesions to the substrate) and membrane ruffles including circular dorsal ruffles which are also F-actin rich but which occur on the dorsal aspect of the plasma membrane [23].

Cell adhesion sites are locations where the cell membrane is in close apposition to the substrate and were originally identified using electron microscopy [24, 25] and interference reflection microscopy [26, 27]. Cell adhesions include a spectrum of immature to mature structures with varied molecular composition [28] including focal complexes (also known as focal contacts) and focal adhesions (also known as adhesion plaques). Proteins that label focal complexes and focal adhesions include integrins, vinculin, paxillin and F-actin and α-actinin [3, 29–31].

Changes in adhesion occur through dissolution and restructuring of focal complexes and focal adhesions in response to PDGF [3] and during other morphologically driven processes such as spreading and motility [32]. We showed that Huntingtin re-localizes to the plasma membrane in response to growth factor stimulation in human fibroblasts and clonal striatal cells and interacts with PI(3,4,5)P3 [33]. These findings prompted us to ask whether Huntingtin is important for actin dependent morphology changes in fibroblasts.

Here we show that Huntingtin is required for maintenance of adhesion and normal cell morphology changes in response to growth factor stimulation and that Huntingtin co-localizes with α-actinin and regulates its stimulus dependent localization at membranes. α-actinins are actin binding proteins that bundle and crosslink actin filaments in both contractile and non-contractile cells [34, 35] and link actin filaments to β1 integrin in focal complexes and focal adhesions [3, 30, 31]. Mutations in α-actinin genes are associated with human diseases [36]. In brain, actin and α-actinin-2 are enriched in neuronal dendritic spines and function in regulating spine shape and stabilizing proteins at postsynaptic membranes [37]. We show that immunoprecipitation of exogenous FLAG-tagged Huntingtin can co-precipitate exogenous GFP-tagged α-actinin-2 and map a potential association domain in Huntingtin.

## Materials and methods

### Cell culture

Primary human fibroblasts were obtained from Coriell Cell Repository and used within 10 passages. Cell line number and CAG repeat on each allele are as follows: GM08399, 17/19, Control1; GM04837, 44/47, HD1; GM04855 20/50, HD2; GM03440B, not determined, “unaffected” control (Control 2). CAG number reported here was determined by Sanger sequencing after the original collection of tissue and provided by David Houseman (personal communication). Cells were cultured using recommended medium containing 15% FBS in MEM supplemented with 2x essential amino acids, 2x non-essential amino acids, 2x vitamins plus 1x glutamax (all from ThermoFisher/Invitrogen). Human embryonic stem cells (hESCs) were obtained from WiCell (WA09, unaffected control; Provider Scientist: James A. Thomson, University of Wisconsin -Madison). ESCs were grown in feeder free conditions in M2 defined medium (GENEA) on Collagen coated plates (6 well dishes, # 354400) that were further coated with 10 μg/ml Fibronectin overnight at 37 °C and crosslinked with UV for 15 minutes. Cells were passage using Accutase (Invitrogen). All procedures were performed with approval from Partners Biosafety and Partners Human Embryonic Stem Cell Research Oversight Committees (ESCRO# 2015-01-02). Prior to PDGF experiments, primary fibroblasts were made quiescent by incubating in serum-free medium for 48 hours before the addition of 100ng/ml PDGF-BB (Biosource) for 15 minutes at 37°C. The maintenance medium was supplemented with both essential and non-essential amino acids. No evidence of increased autophagy was observed in cells serum starved in this enriched medium. The siRNAs targeting exon1 of Huntingtin (named E1-4) or GFP were previously described [38]. The target sequence for GFP does not exist in the human genome and is thus a non-targeting sequence. We prefer validated non-targeting sequences to scrambled since scrambled versions could still be present in the genome and cause off- target effects. SiRNAs were introduced at 20 nM using 0.8 μL Dharmafect reagent per 200 μL serum-free media to cells plated at 2500 cells/coverslip on 12mm glass coverslips coated with poly-L-lysine 48 hours after plating in complete media. SiRNA-transfected cells were maintained in serum-free media containing siRNA for 48 hours then incubated for 15 min in serum-free media containing 100 ng/mL PDGF-BB (Gibco) or serum-free media alone (control). The siRNA targeting GFP conjugated to Cy3 and transfected using the same conditions to assess transfection efficiency.

### Antibodies and cell stains

**For western blot:** polyclonal anti-Huntingtin 1–17 Ab1(1μg/ml) [39], α-actinin-2 (1:2000; ab68167; Abcam; specific [37], α-actinin (2μg/ml; ab18061; Abcam; may cross react with isoforms 1–4), GAPDH (1:6000, MAB374, Millipore). **For fluorescent labeling:** Anti-Huntingtin Ab2527 and Ab1173 were previously described [40, 41]. Both are rabbit polyclonal made against peptides covering aa 2527–2547 and aa 1173–1196 of Huntingtin, respectively and were used at 3 μg/ml. The immunogen peptides consisted of the following amino acids: “ySCLEQQPRNKPLKALDTRFGR” and “ySLTNPPSLSPIRRKGKEKEPGEQA”. Anti-α-actinin (1:500; ab18061; Abcam; may cross react with isoforms 1–4), vinculin (1:100; Sigma). Secondary antibodies include Cy3 goat anti-mouse secondary antibody (1:500; Jackson Immunoresearch); Cy3 goat anti-rabbit secondary antibody (1:500; Jackson Immunoresearch); Bodipy Green goat anti-mouse secondary antibody (1:500; Invitrogen). Stains include Alexa Fluor 488-Phalloidin and Rhodamine-Phalloidin (1:500; Molecular Probes) for F-actin, and DAPI (1:500; Sigma) or Hoechst stain (1:1000; Molecular Probes) for nuclei. For peptide blocking experiments, 3 μg/ml Ab2527 in 4% Normal Goat Serum/PBS was incubated overnight with 30 μg/ml peptide (above) overnight at 4°C or with an unrelated peptide (yEPGDQENKPCRIKGDIGQST).

### Immunofluorescence, proximity ligation assay (PLA) and confocal microscopy

Cells were washed once with 1X PBS at 37°C containing 2 mM CaCl_2_ and 1 mM MgCl_2_ and fixed for 15 minutes in 4% PFA/4% sucrose in 1X PBS also containing 1 mM MgCl_2_ and CaCl_2_**.** Cells were washed twice with 1X PBS, permeabilized for 30 minutes in 0.1% Triton, washed twice with 1X PBS then blocked 1 hour in 4% NGS (Jackson Immunoresearch). For experiments using DiI, no detergent was used. Cells were incubated in primary antibodies or Alexa Fluor 488 Phalloidin or rhodamine-Phalloidin (1:500; Molecular Probes) in 4% NGS overnight at 4°C. Cells were washed twice with PBS then incubated 1 hour at RT in secondary antibodies and 1:500 DAPI (Sigma). Cells were washed twice with PBS then mounted in ProLong Gold Antifade Mountant (Molecular Probes). For peptide blocking experiments, peptide antibody mixtures (see above) were incubated with fixed and permeablized cells overnight at 4°C, washed 3x with PBS, then incubated with rhodamine-phalloidin (1:1000) for 2 hours. Cells were then washed and incubated with Alexa Fluor 488 secondary anti-rabbit and Hoechst stain for 2 hours, washed with PBS and mounted with ProLong Gold. A Nikon Eclipse TE300 fluorescent microscope equipped with a Biorad Radiance 2100 confocal laser scanner was used to obtain images with a Nikon Plan Apo 60X or 100X oil objective (numerical aperture 1.4). Sequential images for each fluorochrome were acquired using Lasersharp 2000 software and merge in Adobe 7.0. For pixel intensity analysis, confocal settings were kept constant among samples. Numbers of vinculin-positive adhesions per cell were counted in control and homozygous HD 47/44 cells (the HD line which did not have Atypical Type 8 cells). Vinculin-positive adhesions were counted in control and HD fibroblasts in serum-containing medium 24 hours after plating on poly-L-lysine coated glass coverslips. Sharp structures intensely labeled for vinculin were defined as mature adhesion plaques (FAs); slightly oval or more amorphorous/granular vinculin-labeled structures were also counted [28]. 10 cells per coverslip were counted over 3 coverslips and combined (n = 30). To quantify amount of immunofluorescence for α-actinin on the plasma membrane in fibroblasts, three regions of interest (ROI) at membrane ruffles per cell were selected on confocal images by an observer blinded to conditions and average intensity measured using ImageJ. The average of the three ROIs was calculated per cell; 10 cells per coverslip were analyzed from 3 coverslips per condition (biological replicates). Proximity ligation assays (PLA) were performed using the Duolink kit (Sigma) according to instructions and using the same primary antibody concentrations used for immunofluorescence for polyclonal Ab1173 and monoclonal α-actinin-1. As a control for the PLA reaction, polyclonal anti-PSD95 was used at the same concentration as Ab1173 with α-actinin-1 and resulted in <10 foci per cell. Similar results were used when Ab1173 and a monoclonal antibody to Snap25 (also absent from fibroblasts) used at the same concentration as α-actinin-1were used in the PLA reaction. To quantify PLA results, 10 confocal fields of cells from each reaction were acquired by confocal microscopy using Kalman and identical settings (laser, gain, 1.2 aperture, 60x oil objective) among treatment groups. Foci in cell bodies were counted in Image J by an observer blinded to conditions.

### Morphology analysis

For morphology analysis in fibroblasts, siRNA treated cells stained with Alexa Fluor 488 Phalloidin were viewed at 60X with an oil objective by an observer blinded to conditions. Cell shapes were categorized as “Typical” morphology (Types 1–3) which were usually observed in normal control cultures normal cultures treated with PDGF-BB or “Atypical” morphology (Types 4–7). Types 1–3 were flat, could be polarized or non-polarized in shape and had well spread cytoplasm. Type 1 had stress fibers. Types 2 and 3 had few or no stress fibers, and some with ruffled membranes or lamellipodia (Type 2). “Atypical” morphology (Types 4–7) had no stress fibers. Type 4 cells were moderately spread and have high proportion of ruffling of the plasma membrane. Type 5 cells were not well spread with some apparent retraction of the cytoplasm toward the nucleus. Type 6 cells were rounded with short adherent extensions off the plasma membrane and numerous small sites of adhesion at the periphery. Type 7 cells were fully rounded with no apparent adhesions, tenuously adherent. The sums of cell types were derived from 15 fields per coverslip x 3 coverslips (biological replicates) per condition. The total cell numbers counted for each group were: Mock, 219; Mock +PDGF, 271; GFP siRNA, 269; GFP siRNA +PDGF, 272; Huntingtin siRNA, 203, Huntingtin siRNA+ PDGF, 161. Total numbers in each category were compared using Pearson’s chi square test and Post-hoc chi square test corrected for multiple comparisons. The H_0_ (Null hypothesis) was “The morphology of cells treated with siRNA targeting GFP are the same as cells treated with siRNA targeting Huntingtin”. Changes in morphology with Huntingtin depletion were qualitatively observed three separate siRNA experiments and quantified in one experiment from 3 biological replicates. Type 8 cells found in HD cultures were bipolar spindle-shaped.

### SDS PAGE & western blots

Proteins were separated by SDS-PAGE using 3–8% Tris-acetate gels or 4–12% Tris-bis gels (Life Technologies) then transferred to nitrocellulose using iBlot semi-dry transfer apparatus (Life Technologies) or Trans-Blot Turbo (BioRad) and developed using SuperSignal West Pico Chemiluminescent Substrate (Thermo Scientific). Statistical analysis was performed using Microsoft Excel or Prism (GraphPad Software). Pixel intensity quantification was performed using ImageJ (NIH) and signal for Huntingtin was standardized to signal for GAPDH. Some blots were stripped before re-probed using Reblot (Millipore) per manufacturer’s instructions. The stripping buffer is pH 13.3.

### Immunoprecipitations

For association of exogenous proteins and deletion analysis, HeLa cells were transiently co-transfected with 36 μl Superfect (Qiagen) and a total of 5 μg plasmid DNA (2.5μg ACTN2 plus equimolar amounts of pCDNA3.1 either empty or containing FLAG-tagged HTT cDNAs of various lengths to a total of 1.31 pmol). Amounts pCDNA3.1 Flag HTT plasmids were adjusted to equimolar concentrations calculated by the varied HTT sequence. ACTN2-pEGFP was a gift from Johannes Hell (Addgene plasmid # 52669) [42]. Cells were lysed with IP buffer with inhibitors (50 mM Tris, 150 mM NaCl, 1 mM MgCl_2_, 1 mM NaF, 1 mM Na_3_VO_4_, 1% NP 40, 10% glycerol pH 7.4 and containing protease inhibitors (complete midi EDTA-free, Roche)) then incubated with 30 μl M2-ani-FLAG sepharose beads (Sigma) for 3 hours at 4°C. FLAG immunoprecipitates were washed 3 times with IP buffer followed by 2 washes with a higher stringency buffer (50 mM Tris, 250 mM NaCl, 1% NP40, 5mM EDTA, pH 7.2) to reduce background binding of actinins to the beads. Eluates were separated by SDS-PAGE and analyzed by western blot. For FLAG-HTT399-3144 co-expressed with GFP-ACTN2 or controls, HeLa lysates were prepared in the same IP buffer. 375 μg of lysates, first precleared with protein G-Sepharose 4-flastflow (GE Healthcare), were incubated overnight at 4°C with 3μl anti-Huntingtin monoclonal antibody mAb2166 (Millipore). Lysates were incubated 2 hours with protein-G-Sepharose then immune complexes were washed 3x with IP buffer and 1x in PBS. Immunoprecipitated proteins were eluted in 50μl sample buffer and analyzed by SDS-PAGE and western blot for ACTN2. Blot was stripped then re-probed using anti-FLAG M2 (Sigma).

### Statistical analysis

Blinded observers performed pixel intensity analysis and morphology analyses on encrypted images or slides. Paired t-tests were used for comparisons of the same cell line treated or untreated and unpaired t-tests were used for comparisons between two cell lines or two animal genotypes. For comparisons among multiple samples ANOVA and Tukey’s HSD posthoc tests were performed in R or using GraphPad Prism.

## Results

### Reduction of endogenous Huntingtin alters adhesion and morphology of primary human fibroblasts

**Fig 1:**
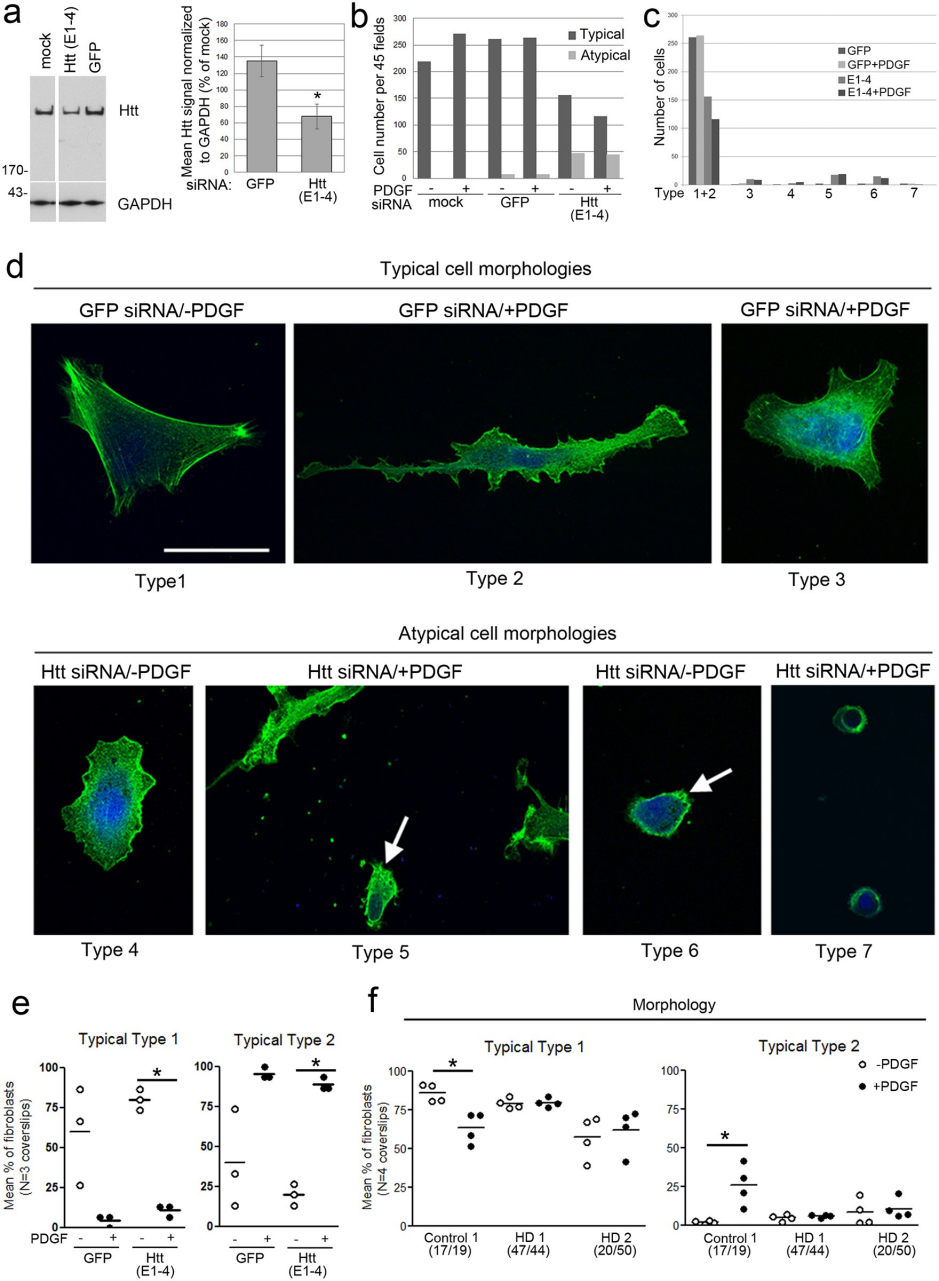
Acute loss of endogenous Huntingtin changes cell morphology. **(a)** Western blots show Huntingtin signal is reduced in Control 1 fibroblasts treated with siRNA targeting exon1 of Huntingtin (E1-4) compared to cells treated with siRNA against GFP (a control siRNA) or mock treated cells. Full blots are shown in S1a Fig. Graph shows mean ±SD for Huntingtin (Htt) total pixel intensity standardized to GAPDH as percent of mock treated cells from western blots (*p<0.01, n = 4 biological replicates, unpaired t-test compared to GFP. **(b)** Graph shows total number of Typical and Atypical cells identified using F-actin labeling. Results are shown for mock (no siRNA plus transfection reagent), siRNA targeting GFP or siRNA targeting Huntingtin (E1-4) and in the absence or presence of 100 ng/ml PDGF for 15 min. Total cell number counted for each group was mock, 219 cells; mock + PDGF, 271 cells; GFP siRNA, 269 cells; GFP siRNA + PDGF, 272 cells; Huntingtin siRNA, 203 cells, Huntingtin siRNA+ PDGF, 161cells (3 coverslips per group). Populations were compared using Pearson’s chi square test (p = 1.25 E-22), Mantel-Haenszel analysis and showed a highly significant change in cell shape in response to treatment with Huntingtin (E1-4) when controlled for PDGF treatment (p<2.2e-16; confidence interval is p<0.05) and no change in cell shape in response to PDGF treatment when controlled for treatment with Huntingtin (E1-4) (p = 0.4103). **(c**) Graph shows breakdown of cell counts for each category shown in Fig 1b and described in d. **(d)** F-actin labeling in primary fibroblasts. Sample of representative images of cells with “Typical” morphologies (Types 1–3) found in control cultures and “Atypical” morphologies (Types 4–7). “Typical” morphology (Types 1–3) have flat, polarized or non-polarized shape, have cytoplasm is well-spread with stress fibers (Type 1) or without stress fibers (Types 2 and 3), and some with ruffled membranes or lamellapodia (Type 2). “Atypical” morphology (Types 4–7) are moderately spread and have high proportion of ruffling of the plasma membrane (Type 4), are not well spread with some apparent retraction of the cytoplasm toward the nucleus (Type 5, arrow), are rounded with short adherent extensions off the plasma membrane (Type 6, arrow), or are fully rounded with no apparent adhesions (Type 7). Cells stained with Alexa-Phalloidin. Scale bar = 50 μm and applies to all images in d. **(e)** SiRNA treated Control 1 cells were assessed for morphology Types 1 and 2 in serum starved or stimulated with 100 ng/ml PDGF for 15 minutes. Graph shows mean percent ±SD. *<0.05, paired t-test, n = 3 coverslips. (f) Control 1 (17/19) and two HD human fibroblast lines (HD1 47/44 and HD2 20/50) were assessed in parallel for morphology types described in d. Cells were serum starved or stimulated with 100 ng/ml PDGF for 15 min. Graphs show mean percent ±SD for number of Type 1 (left) and Type 2 (right)’ Typical cells and Type 8 Atypical cells are shown in S2c Fig. A significant loss of Type 1 cells and a gain of Type 2 cells occurs in Control 1 fibroblasts with stimulation reflecting membrane ruffling (Two-way ANOVA and Bonferroni posthoc test, *p<0.05, n = 4 coverslips). Numbers in parentheses on x-axis indicate CAG repeat length for each Huntingtin allele in each cell.

To determine the effect of Huntingtin on cell morphology, control fibroblasts (Control 1 cells, 17/19 CAGs) were treated with small interfering RNA (siRNA) to lower Huntingtin protein levels and analyzed by fluorescence microscopy for filamentous actin (F-actin) labeling using Alexa-phalloidin with and without stimulation. At 48 hours post-transfection, treatment with a siRNA targeting Huntingtin (E1-4) significantly lowered Huntingtin by 50% compared to a control siRNA targeting GFP (Fig 1a and S1a Fig). A transfection of Cy3-labeled GFP siRNA in the same fibroblasts cells (Control 1) using the same conditions showed 79±0.6% of cells were labeled compared to 0% labeling in the MOCK condition (no siRNA plus transfection reagents) (S1c Fig, n = 3 coverslips). Lowering Huntingtin with siRNA in the presence or absence of PDGF changed the morphology of fibroblasts in the cultures to produce some cells with an atypical morphology not seen in control untreated or mock treated cells. To quantify changes in morphology, we devised a classification system described in Methods and in Fig 1. Cells were classified as having Typical (Types 1–3) and Atypical (Types 4–8) morphologies. Lowering Huntingtin reduced the number of cell types with Typical morphologies (flat, well-spread) normally observed in serum-starved conditions and increased the numbers of cells with Atypical morphologies (less flat, rounded) (Fig 1b, 1c and 1d). Atypical rounded cells (Type 7) had cytoplasm retracted toward the nucleus and were easily lifted off the culture dish with washes suggesting a reduction in adhesion. Treatment with PDGF for 15 minutes further decreased the number of cells with Typical morphologies in E1-4 treated cells and reduced total cell number (Typical + Atypical) compared to control medium, indicating a loss of adhesion (Fig 1b). A similar profile of increased Atypical cells with Huntingtin siRNA compared to GFP siRNA occurred when data is plotted as a percent of the total cells counted for each group (S1d Fig). We probed lysates from the siRNA treated cells for active Caspase 3 to look for evidence of apoptosis. No evidence of proteolysis of Caspase 3 was observed (S1b Fig). Cells with Typical morphologies (Types 1 & 2) were compared for evidence of changes in membrane ruffling to assess their response to PDGF [23]. We observed a significant decrease in Typical Type 1 cells for Control 1 cells treated with Huntingtin siRNA E1-4 and an increase in Typical Type 2 cells for Control 1 cells treated with Huntingtin siRNA E1-4 and GFP siRNA (Fig 1e). These observations indicate an expected increase in ruffling and morphology change in response to PDGF suggesting that cells with Typical morphologies fully respond to PDGF and increased ruffling to similar levels with Huntingtin lowering. These findings show that Huntingtin is necessary for maintaining normal cell morphology and is required for cell adhesion following stimulation with a growth factor that initiates changes in cell morphology. Huntingtin is not necessary for initiating the morphology response to PDGF, however.

### Mutant Huntingtin disrupts growth factor induced cell morphology changes

To determine if the presence of the HD mutation might impact dynamic cell morphology, control and HD human fibroblasts were treated with PDGF and assessed for morphology using the same classification system. In control fibroblasts (Control 1, 17/19 CAGs), we observed a significant decrease in Typical Type 1 cells and an increase in Typical Type 2 cells reflecting an expected increase in ruffling and morphology change in response to PDGF (Fig 1f). The same response of a significant decrease in Typical Type 1 cells and an increase in Typical Type 2 cells occurred in another control fibroblasts cell line (Control 2) (S2a Fig). In contrast, no change in cell morphology was observed in heterozygous HD2 (20/50) or homozygous HD1 (44/47) cell lines (Fig 1f), indicating a failure to respond to PDGF. Most HD cells had Typical morphologies, however, we did observe a new different cell morphology in one HD cell line (HD 20/50) compared to control cells, Atypical Type 8 (S2b Fig), which were not well spread or loosely adherent, but were long and spindly. The numbers of these Type 8 cells comprised about 19.4% of HD 20/50 cells and did not change with PDGF treatment **(**S2c Fig). Type 8 cells were also very low in cultures of Control 2 cells (S2d Fig). Altogether, these data show that reduction of normal Huntingtin does not inhibit the initial morphology change in response to PDGF, but results in a loss of adherent cells. In contrast, the presence of the HD mutation inhibits changes in morphology in response to PDGF.

### Huntingtin co-localizes with actin stress fibers and is present at nascent focal adhesion sites

**Fig 2:**
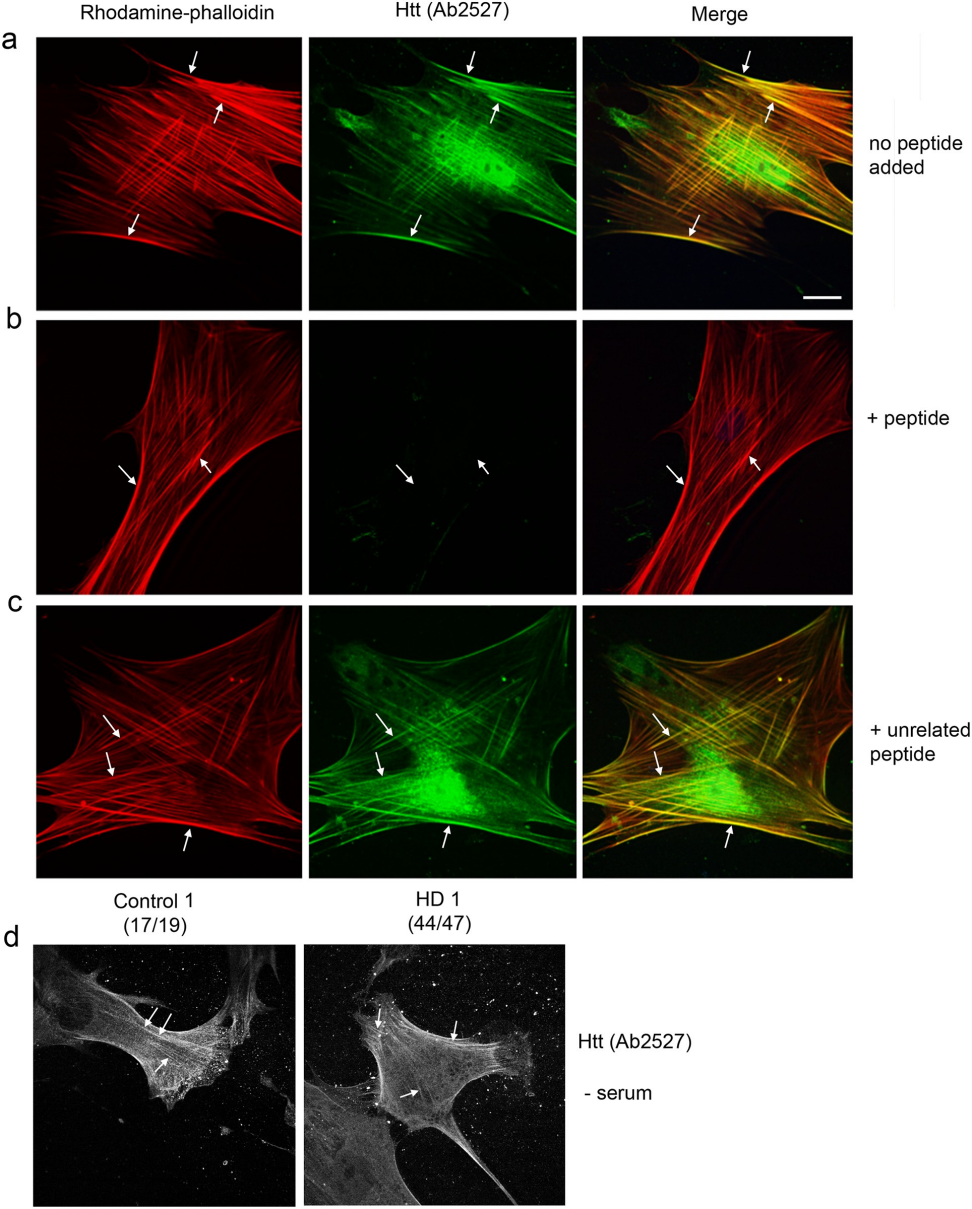
Huntingtin co-localizes with F-actin stress fibers in normal human fibroblasts. **(a)** Immunofluorescence and confocal microscopy performed with anti-Huntingtin (Htt) antibody Ab2527 (Green) and rhodamine-phalloidin (Red) to visualize F-actin in serum-starved Control1 cells. Co-localization (Yellow) is observed in merged images at right. Immunostaining for Ab2527 is coincident with F-actin stress fibers (arrows). Small vesicles and nuclei were also labeled. (b) In parallel cells were immune-stained with Ab2527 pre-incubated with ten-fold mass of specific blocking peptide. The presence of the blocking peptide eliminated filamentous, small vesicle and nuclear staining. Arrows show where phalloidin-positive actin stress fibers are present in the cell. (c) Cells were immune-stained with Ab2527 pre-incubated with ten-fold mas of an unrelated peptide. The normal staining for Ab2527 including filamentous (arrows), vesicular and nuclear staining are evident. (d) Immunostaining with Ab2527 performed in parallel in Control1 and HD1 cells. Both control and HD cells showed normal filamentous staining (arrows). Scale bar in a = 5 μm and applies to all images.

Our results above showed that reducing Huntingtin protein affects actin dependent morphology changes. We previously reported that by immunohistochemistry the anti-Huntingtin polyclonal antibody, Ab2527, labeled vesicles, a perinuclear vesicular compartment and nuclei in human fibroblasts [41]. Although not a focus of our previous study, we also noticed stained structures more peripheral in the cell consistent with large bundles of F-actin called stress fibers. To demonstrate co-localization of Huntingtin and actin we performed double-labeling for rhodamine-phalloidin, which binds to polymerized F-actin, and for Huntingtin with Ab2527 in serum-starved Control 1 fibroblasts (Fig 2a). Huntingtin immunostaining was coincident with phalloidin-positive fibers transiting along the plasma membrane and also occurred on perinuclear vesicles and in nucleus as previously described [40, 41]. When pre-incubated with a blocking peptide containing the immunogen, the staining for Ab2527 was greatly reduced and staining co-incident with stress fibers and staining in the nucleus and in the perinuclear compartment were eliminated (Fig 2b). In contrast, pre-incubation of Ab2527 with an unrelated peptide had no effect on binding of Ab2527 or phalloidin and normal staining patterns were observed **(**Fig 2c). Ab2527 immunofluorescence was very similar in control cells and HD cells grown in serum-free medium (Fig 2d). Brefeldin A treatment, which releases many proteins from trans Golgi network (TGN) derived vesicles, had no effect on Huntingtin immunoreactivity along phalloidin-positive cables but did reduce Huntingtin immunoreactivity on perinuclear vesicles (S3 Fig). This finding suggested that Huntingtin associates directly with stress fibers and to brefeldin A-sensitive small TGN-derived vesicles.

**Fig 3:**
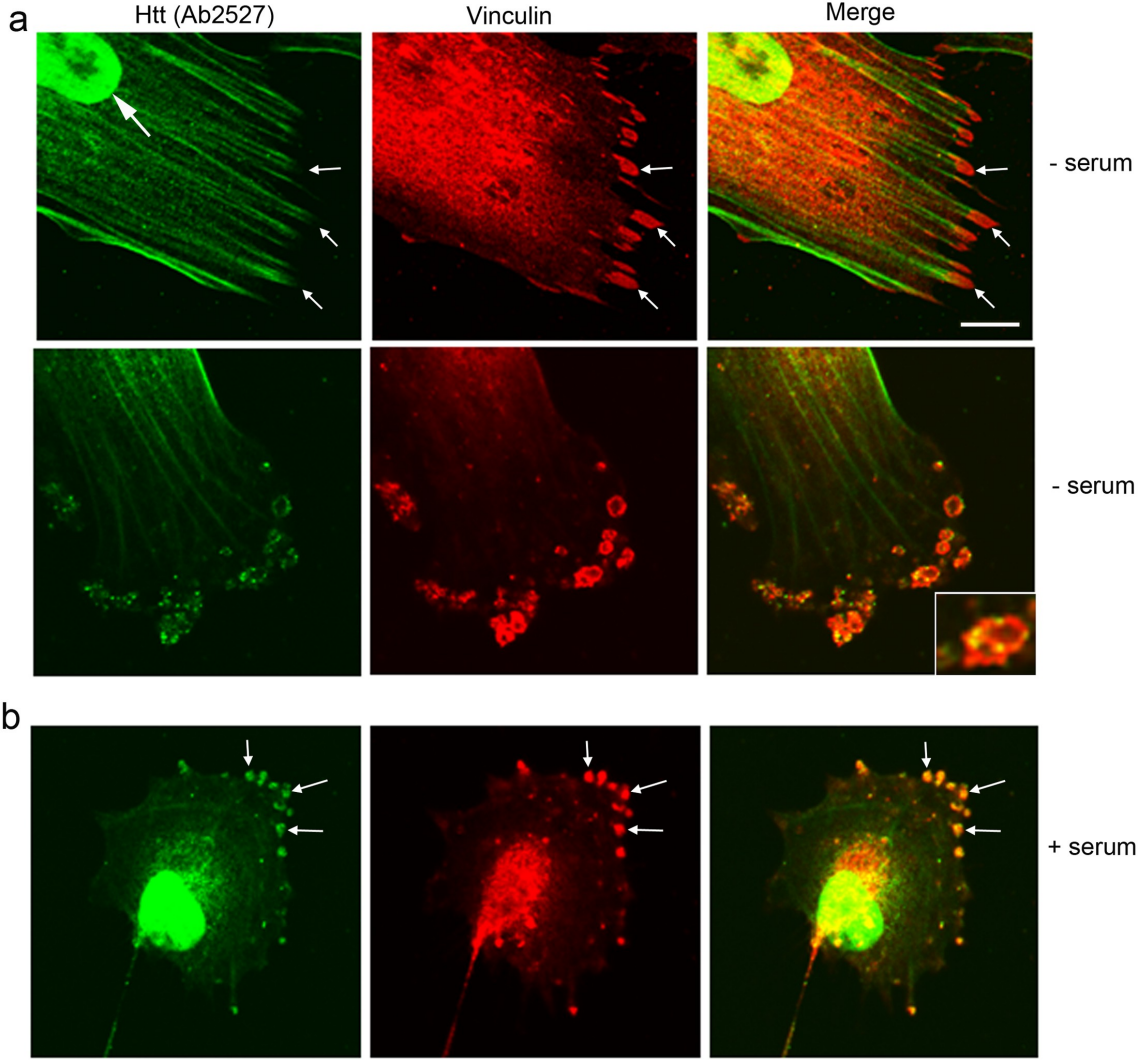
Huntingtin can co-localize to vinculin-positive adhesion structures. Double-label immunofluorescence with anti-Huntingtin antibody Ab2527 (Green) and anti-Vinculin (Red). **(a)** In serum starved cells, Huntingtin immunoreactivity occurs along stress fibers leading into large Vinculin-positive adhesion plaques (top row, arrows), from which Huntingtin is excluded. In cells containing less prominent stress fibers (bottom row), small foci of staining for Huntingtin with Ab2527 was present in Vinculin-positive adhesion structures that appeared less well-organized than those depicted in a suggesting immature or developing adhesion plaques (inset). **(b)** In cells grown with serum with no stress fibers and with focal contacts, Huntingtin labeled with Ab2527 and Vinculin co-localize (arrows in merged). Scale bar in a = 5 μm and applies to all images.

Vinculin is a marker of the early formation of focal complexes and is present throughout their maturation to focal adhesions [29, 43, 44]. Double-label immunofluorescence showed Huntingtin detected with Ab2527 was absent from mature vinculin-positive adhesion plaques at the ends of stress fibers in quiescent (serum-starved) cells (Fig 3a). In a cell containing some stress fibers but less well organized/mature vinculin-positive adhesion plaques, staining for Ab2527 showed small foci of immunostaining in the adhesion plaque (Fig 3a, bottom panel and inset). In cells that were grown in serum, Huntingtin detected with Ab2527 co-localized with vinculin in structures at the periphery of cells that lacked stress fibers (Fig 3b). Confocal images were acquired at the level of the ventral aspect of the cells where the plasma membrane is in contact with the substrate suggesting these are focal complexes (Fig 3b**, arrows).** Overlap was about 30–50% within these structures. More diffuse staining for Huntingtin and vinculin occurred within the cell body and some co-localization of signal occurred in the perinuclear area, however the signal was saturated in this region since the image was taken to visualize the peripheral structures **(**Fig 3b**).**

**Fig 4:**
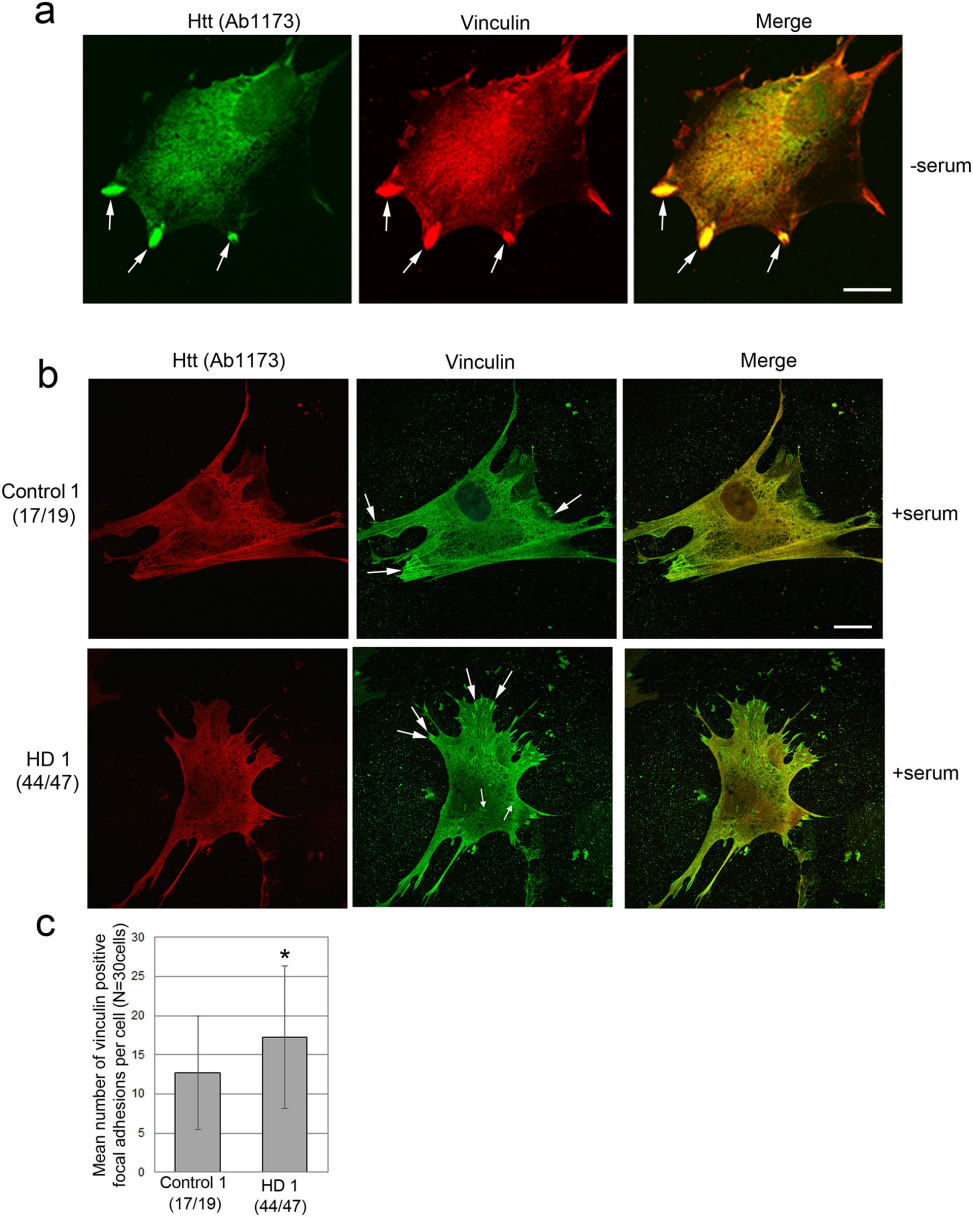
Huntingtin detected with Ab1173 can localize to vinculin-positive adhesions. Immunofluorescence for Huntingtin with Ab1173 in Control 1 and HD1 human fibroblasts is similar, but Vinculin-positive adhesion plaques are increased in HD1 cells. **(a)** Double label immunofluorescence with Huntingtin labeled with Ab1173 (Green) and Vinculin (Red) shows colocalization in focal adhesions in serum starved cells. Scale bar in a = 5μm. Shown are representative images. **(b)** Double-label immunofluorescence and confocal microscopy of cells grown in normal serum containing medium shows similar staining of anti-Huntingtin Ab1173 in Control 1 and HD1 human fibroblasts. Staining was diffuse through the cytoplasm with lower levels in the nucleus (red). Staining for Vinculin (green) was diffuse throughout the cell with accumulations in focal adhesions (arrows) at the periphery of the cell. Scale bar in b = 5μm. **(c)** In control plus serum conditions, HD1 47/44 fibroblasts had significantly increased numbers of vinculin-positive focal adhesions per cell compared to Control1 (17/19) cells (17.2 ± 9.1 per cell for HD (47/44) compared to 12.7 ± 7.3 for Control (17/19) cell; p = 0.037, unpaired t-test, n = 30 cells).

Using anti-Huntingtin antibody Ab1173 there was also co-localization with vinculin in structures that looked like focal adhesions (Fig 4a**).** The amount of overlap with vinculin as within these focal adhesions was at least 70%. There was also some overlap between Huntingtin and vinculin immunofluorescence in the nuclear and perinuclear regions. No other anti-Huntingtin antisera beside Ab2527 and Ab1173 labeled vinculin positive structures suggesting that a specific conformation of total Huntingtin is co-localized with vinculin. No gross changes for Ab1173 staining were observed previously in HD fibroblasts compared to controls [40] and we did not observe changes in staining in cells grown in serum (Fig 4b). We queried whether changes specifically in focal adhesions might occur in cells expressing mutant Huntingtin. Numbers of vinculin-positive adhesions per cell were counted in Control 1 (17/19) and homozygous HD1 (44/47) cells (the HD line which did not have Atypical Type 8 cells). Sharp structures intensely labeled for vinculin and usually close to the cell periphery were defined as mature focal adhesions (Fig 4b, arrows); slightly oval or more amorphorous/granular vinculin-labeled structures described by others and usually more centrally located within the cytoplasm [28] were also counted (Fig 4b**, small arrows**). Small nascent focal complexes were abundant in lamellipodia in control and HD cells and were not counted. In normal growth medium-containing serum, HD1 47/44 fibroblasts had significantly increased numbers of vinculin-positive mature focal adhesions per cell compared to Control 1 (17/19) cells (17.2 ± 9.1 per cell for HD1 (47/44) compared to 12.7 ± 7.3 for Control 1 (17/19) cell; p = 0.037, unpaired t-test, n = 30 cells) **(**Fig 4c). There was no change in the number of oval, granular vinculin labeled structures. Altogether, these results show that a portion of Huntingtin co-localizes with F-actin and is present during early formation of focal complexes that are positive for vinculin. Furthermore, the results suggest that the presence of mutant Huntingtin may increase cell adhesion by increasing the number of mature focal adhesions.

### Exogenous Huntingtin co-immunoprecipitates with exogenous α-actinin-2

Previous Huntingtin interactomes generated using affinity MS and tandem MS data showed interactions with the actin binding and cross-linking proteins α-actinin-1, -2 and -4 [15, 16]; these interactions were never validated by other means, however. The staining pattern reported for anti-α-actinin antisera was similar to that of actin [45] and to the staining we observed with Ab2527 (Fig 2). To ascertain if the two proteins could interact, we overexpressed FLAG- tagged Huntingtin with GFP-tagged -α-actinin-1 or with GFP-tagged α-actinin-2. Overexpressed GFP-α-actinin-1 was very “sticky” and was detected all precipitation reactions including in control precipitation reactions where specific antibodies were omitted and could not be further evaluated. In contrast, exogenous GFP-α-actinin-2 did not precipitate in control reactions and was selected for further analysis.

**Fig 5:**
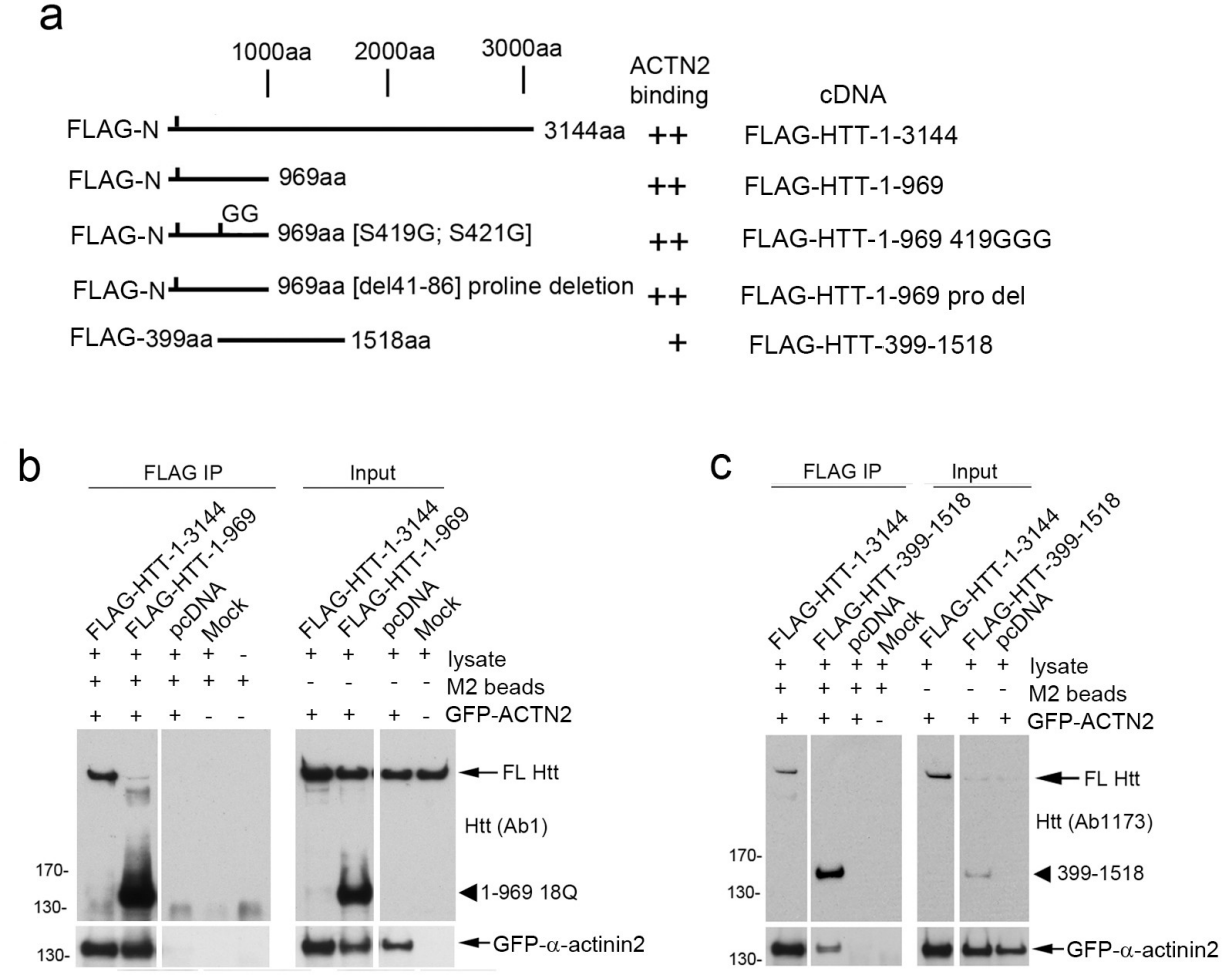
Exogenous α-actinin-2 and Huntingtin associate in lysates from human HeLa cells and deletion analysis maps the interaction region to aa 399–969 of Huntingtin. (**a**) Deletion analysis was performed in HeLa cells using Huntingtin cDNA constructs as depicted co-expressed with a cDNA for GFP-α-actinin-2 (GFP-ACTN2). The CAG length in Huntingtin was 18 (wild-type). Ability to pull down GFP-α-actinin-2 indicated at right. **(b)** Western blots of FLAG immunoprecipitates and inputs. FLAG tagged full length Huntingtin (FLAG-HTT-1-3144) or aa 1–969 (FLAG-HTT-1-969) pulls down GFP-α-actinin-2. (**c**) FLAG tagged full length Huntingtin (FLAG-HTT-1-3144) or aa 399–1518 (FLAG-HTT-399-1518) pulls down GFP-α-actinin-2. The minimal shared region was aa 399–969 of Huntingtin. Full blots for b and c are shown in S5 Fig.

To determine if the two proteins interact and to map the domain in Huntingtin which interacts with α-actinins, HeLa cells were transiently co-transfected with expression plasmids containing FLAG-tagged HTT deletion cDNA constructs (Fig 5a, 5b and 5c**; full blots in** S4 Fig) and with GFP-α-actinin-2 cDNA. Blots of FLAG pulldowns were probed with anti-Huntingtin antibody Ab1 then re-probed with an antibody specific to α-actinin-2 (ab68167). The ratio of co-immunoprecipitated FLAG-HTT1-3144 and GFP-α-actinin 2 was highly consistent in 3 experiments. Co-immunoprecipitation experiments with anti-FLAG antibody showed that GFP-α-actinin-2 interacts in a region within aa 399–969 of Huntingtin (Fig 5b and 5c). The protein product of FLAG-HTT399-1518, interacted with GFP-α-actinin-2 but to a lesser degree than full length Huntingtin (FLAG-HTT1-3144) or the FLAG-HTT1-969. This reduced interaction could be due to altered folding properties of this internal protein fragment or because the full binding site contains additional amino acids N-terminal to aa 399 in Huntingtin. No GFP-α-actinin-2 was pulled down in control reactions containing anti-FLAG sepharose beads and lysates from cells which overexpressed α-actinin-2 but no FLAG-Huntingtin construct (Fig 5b**,** lane 3 **and** Fig 5c**,** lane 3). We also performed an immunoprecipitation with mAb2166 against Huntingtin to isolate FLAG-HTT399-3144 because although the cDNA for this construct encodes FLAG, the protein is not recognized by M2 during an IP. The product is only recognized by M2 after exposure to a stripping buffer (pH 13.3) and re-exposure to neutral buffer suggesting an epitope recapture process (S4d Fig); GFP-α-actinin-2 also pulldown with FLAG-HTT399-3144. We also tested a construct expressing aa 1–969 of Huntingtin with double point mutations at S419G and S421G. These serines in Huntingtin are subject to phosphorylation by AKT which is also activated by growth factor stimulation and PI 3-kinase. Thus, phosphorylation at these sites might be necessary for interaction with α-actinin-2. We found that the mutations did not affect binding to α-actinin-2 (S4b Fig, 419GGG).

### Huntingtin co-localizes with α-actinin in human fibroblasts and human neurons

**Fig 6:**
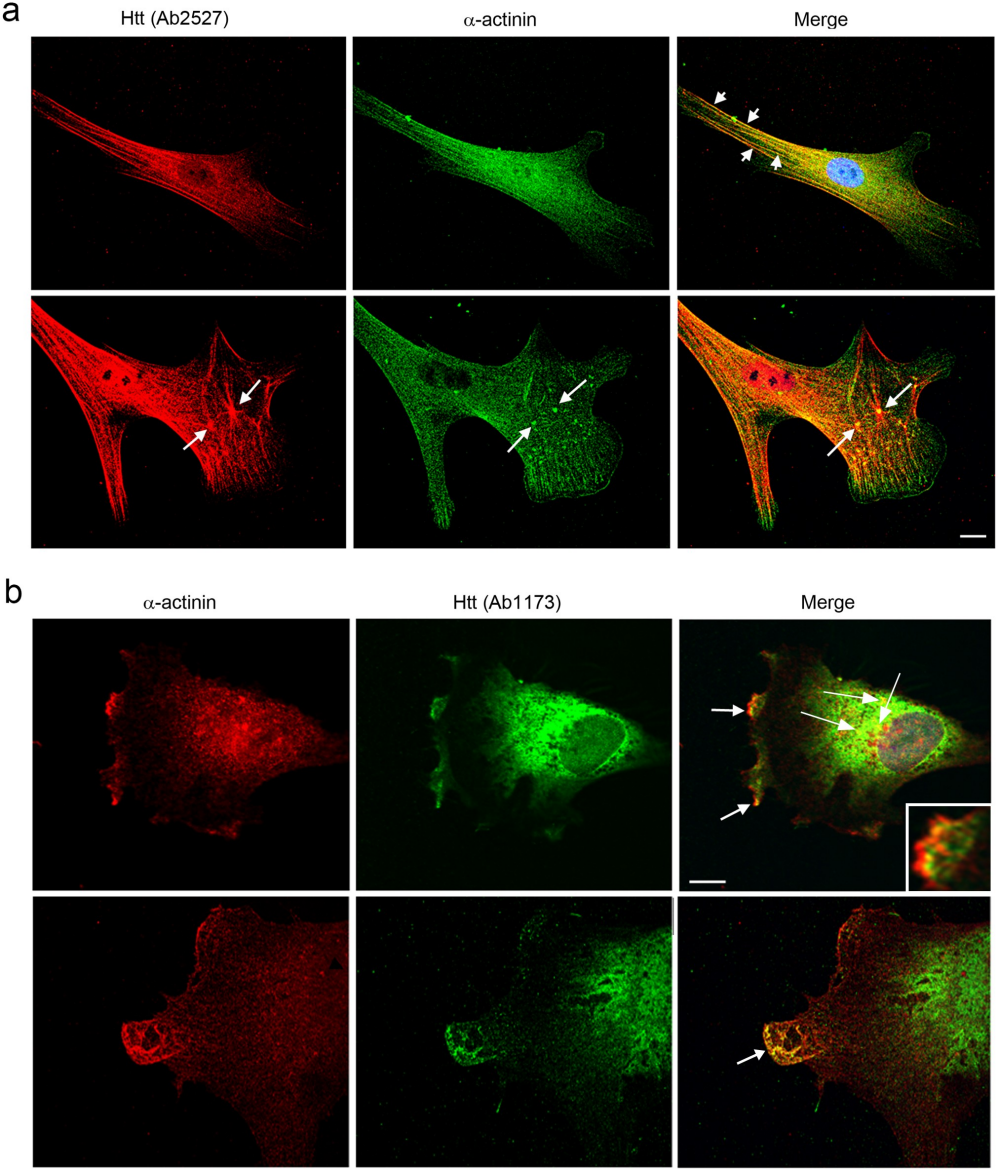
Huntingtin co-localizes with α-actinin-1 in primary human fibroblasts. (**a and b**) Double-label immunofluorescence using two anti-Huntingtin antibodies (Ab2527 and Ab1173) and a monoclonal antibody against α-actinin-1 in human primary fibroblasts. (**a**) Huntingtin detected with Ab2527 (Red) co-localized with α-actinin (Green) at stress fibers in serum-starved cells (top, short arrows). In normal growth medium, rare cells displayed reactivity for Huntingtin detected with Ab2527 on stellate polygonal structures in the cytoplasm consistent with actin microfilament together with reactivity for α-actinin at the vertex (bottom, arrows) as previously described [45]. (**b**) Huntingtin detected with Ab1173 (Green) co-localized with α-actinin-1 (Red) in lamellipodia at ruffled membranes (top panel, arrows and inset) and in protrusions at the leading edge of lamellipodia (bottom panel, arrow). Arrowheads indicate Huntingtin detected with anti-actinin-1 in the cytoplasm at perinuclear sites. Yellow shows co-localization in Merged images. 60x oil objective. Images in a (top panel) and b are representative images. Scale bars = 10μm.

Having established biochemical interaction of Huntingtin and α-actinin-2, we next addressed the sites of co-localization of these proteins in cells. The only antibody specific for α-actinin-2, ab68167, is a rabbit polyclonal antibody as are anti-Huntingtin Ab2527 and Ab1173. This precluded co-localization studies with α-actinin-2. The antibody against α-actinin-1 is a monoclonal antibody but potentially can cross react with all isoforms. Double-label immunofluorescence in human fibroblasts using anti-Huntingtin antisera (Ab2527) together with anti-α-actinin-1 showed extensive co-localization in stress fibers in serum starved cells (Fig 6a**, short arrows**). In some cells, Huntingtin immunoreactivity using Ab2527 stained a large diamond shaped structure surrounding a star-shaped filamentous array in the cytoplasm. α-actinin was present on the vertex of the stellar structure (Fig 6a**, bottom panel, long arrows**). These patterns of staining are similar to those previously described using anti-actin and anti-α-actinin antisera by Lazarides et al., and referred to as stellate polygonal structures [45].

Double label immunostaining with anti-Huntingtin antibody ab1173 and anti-α-actinin-1 showed Huntingtin present in lamellipodia partially overlapping with actinin-1 in membrane ruffles (Fig 6b**, top panel, arrows and inset**), and in protrusions at the leading edge of lamellipodia (Fig 6b**, bottom panel, arrow**). Co-localization of Huntingtin detected with Ab1173 with anti-α-actinin-1 also occurred in the cytoplasm at perinuclear sites (Fig 6b**, arrowheads**).

**Fig 7:**
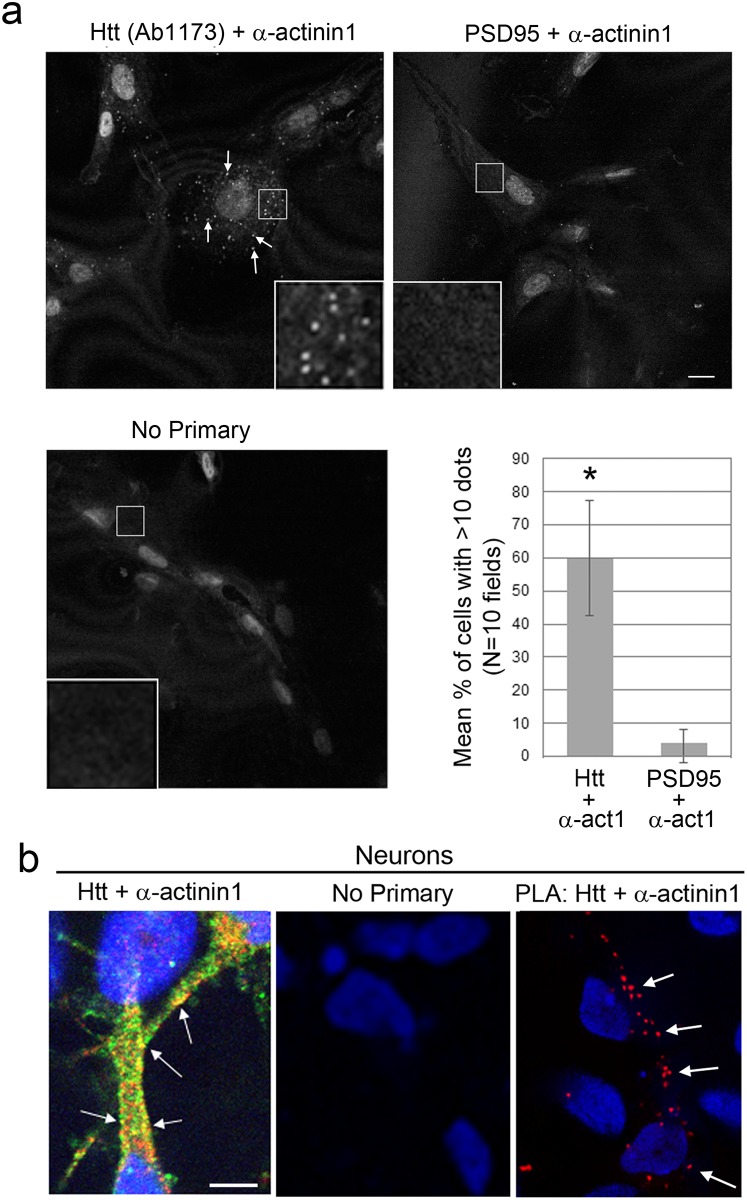
A proximity ligation assay (PLA) produced reaction product when an antibody for Huntingtin (Ab1173) and α-actinin-1 were used. Fluorescence was visualized by confocal microscopy in human Control 1 fibroblasts (**a**) and human neurons (**b**). At top left in a, the fluorescence confocal image shows the reaction product with anti-Huntingtin polyclonal Ab1173 and monoclonal anti- α-actinin-1 in small foci in the cytoplasm. Arrows indicate the reaction product in small foci. Boxed region indicates inset showing dots at increased magnification. At top right in a, confocal image shows low levels of reaction product using polyclonal antibody targeting PSD95 and monoclonal α-actinin-1. At bottom left in a, reaction where primary antibodies were omitted shows no reaction product. At bottom right in a, graph shows mean ± standard deviations percentage of cells with ≥10 foci per cell. N = 10 confocal fields per condition, p<0.0001, unpaired t-test. Images taken with 60X oil objective. In b, the left image shows Merged images of traditional double-label immunofluorescence in a human neuron (left) and no primary antibodies (center). Co-localization in Yellow is observed in the cell body and in patches at the plasma membrane along the primary process in a polarized cell with a neuronal morphology. At right, PLA reaction product (Red, arrows) also shows co-localization in the cell body and along major process. 60x oil objective. Images are representative. Scale bars = 10 μm. Nuclei were stained using Hoechst.

To demonstrate a close molecular interaction, a proximity ligation assay (PLA) with anti-α-actinin-1 and anti-Huntingtin Ab1173 antibodies was performed in human fibroblasts grown both with or without serum and human neurons grown in defined medium. Fluorescent reaction product occurred in discrete puncta in the cytoplasm both within fibroblast cell bodies (Fig 7a**, top left,** cells grown without serum) and rarely at ruffled membranes that were visualized by phase contrast in cells grown with serum (S5a Fig). Low levels of reaction product (<9 foci per cell) occurred with a polyclonal antibody targeting PSD95, an unrelated protein which is absent from fibroblasts and α-actinin-1 (Fig 7a, top right). No reaction product occurred in the absence of primary antibodies (Fig 7a**, bottom left**). Quantification revealed significantly more cells with ≥10 foci per cell when the two specific antibodies Ab1173 and anti-α-actinin-1 were used in the PLA reaction compared to a polyclonal antibody against PSD95 and α-actinin-1 **(**Fig 7a, **bottom right**; p<0.0001, unpaired t-test). This result was consistent with co-localization observed using traditional double-label fluorescence with the same antibody pair (Fig 6b, *top panel*, *arrows*). In human neurons double-label immunofluorescence for Ab1173 and α-actinin-1 revealed co-labeled puncta located within cell bodies and near the plasma membrane (Fig 7b); a PLA reaction in human neurons produced punctate staining like that observed using traditional fluorescence **(**Fig 7b). The number of puncta produced using PLA was far fewer than that observed using double-label fluorescence suggesting only a fraction of the co-localized proteins are interacting at a molecular level. These results support the notion that Huntingtin interacts with α-actinin isoforms in cells including neurons.

### Huntingtin is required for the localization of α-actinin to plasma membrane foci in response to growth factor stimulation in human fibroblasts

**Fig 8:**
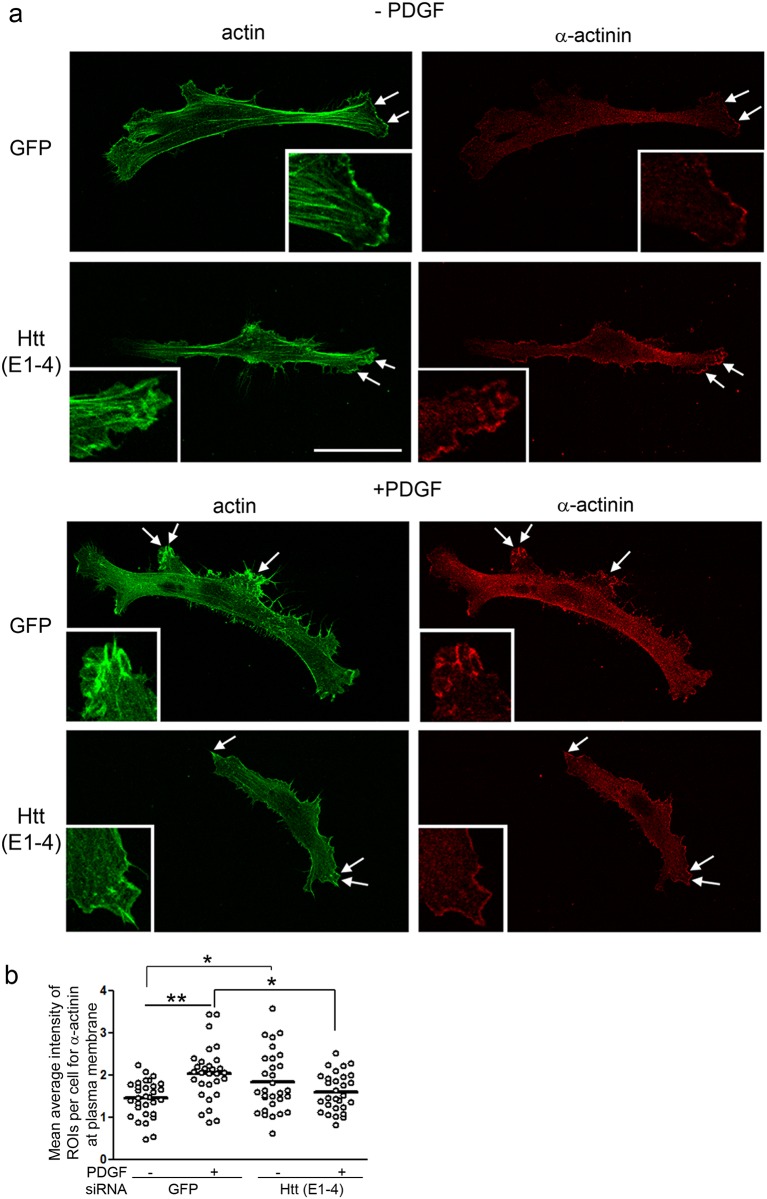
Reduced Huntingtin levels alter localization of α-actinin at the plasma membrane. **(a)** Confocal micrographs of human fibroblasts labeled for F-actin with Alexa-Phalloidin (Green) and α-actinin (Red). Cells were treated with siRNA targeting GFP as a control or E1-4 targeting Huntingtin (Htt). Shown are representative images of cells serum-starved or cells treated for 15 minutes with 100 ng/ml PDGF. Insets show higher magnification of the regions at double arrows. Scale bar = 50 μm. (**b**) Graph shows mean pixel intensity (bar) for α-actinin at membrane ruffles in well spread cells Types 1–3 (see Methods). Statistics were performed by one-way ANOVA and Tukey’s HSD posthoc test, n = 30 cells, 3 coverslips per condition; *p<0.05, **p<0.001.

α-actinin-1 helps promote assembly of dorsal stress fibers at the leading edge during cell spreading and cell migration [46]. We asked if a reduction of Huntingtin protein affected the localization of α-actinin in human fibroblasts using immunofluorescence with an α-actinin antibody and Alexa-phalloidin. To quantify the change in α-actinin localization in typical cells, fibroblasts were imaged by confocal microscopy then pixel intensity analysis for α-actinin was performed in 3 regions of interest (ROI) per cell. Each ROI was placed at membrane ruffles which were visualized using Alexa-phalloidin staining and a sequential scan was performed by an observer blinded to conditions (S5b Fig). Compared to control medium (serum starved), PDGF treatment increased the localization of α-actinin in focal areas at membrane ruffles together with intense staining for F-actin in cells treated with the control siRNA targeting GFP (Fig 8a); pixel intensity analysis also showed a significant rise in the level of α-actinin in ruffled membranes in GFP siRNA treated cells stimulated with PDGF compared to control medium (Fig 8b**;** ANOVA with Tukey’s post-hoc test, **p<0.001, n = 30 cells on 3 coverslips). However, in cells treated with Huntingtin siRNA, α-actinin failed to recruit to focalized sites at membranes in response to PDGF resulting in levels of α-actinin1 that were significantly lower in Huntingtin siRNA treated cells with PDGF compared to GFP siRNA treated cells with PDGF (Fig 8a and 8b**;** ANOVA with Tukey’s post-hoc test, *p<0.05, n = 30 cells on 3 coverslips). In serum-starved conditions (-PDGF), α-actinin immunostaining was increased in membrane ruffles in cells treated with siRNA targeting Huntingtin (E1-4) compared to cells treated with a control siRNA targeting GFP (Fig 8a and 8b; ANOVA with Tukey’s post-hoc test, *p<0.05, n = 30 cells on 3 coverslips). The rounded, Atypical cells previously described with Huntingtin siRNA and PDGF treatment showed intense labeling of α-actinin at the retracting cell periphery (S5c Fig).

These results show that cells with reduced Huntingtin protein fail to recruit α-actinin to foci at the plasma membrane and show a loss of α-actinin immunoreactivity at membrane ruffles upon PDGF stimulation. Overall, these data suggest that Huntingtin regulates stimulus dependent localization of α-actinin on the plasma membrane which may be necessary for focal contact restructuring following cytoskeletal reorganization.

## Discussion

In this study, we found that Huntingtin is required for cellular adhesion and for the normal morphological changes mediated by the actin cytoskeleton in response to growth factor stimulation. Consistent with these findings Huntingtin localized to actin filaments, nascent focal adhesion sites, and membrane ruffles in primary human fibroblasts, which are an established cell model for studies of actin cytoskeleton dependent morphology. In our previous work using transformed cell lines, no obvious changes in morphology were observed by overexpressing constructs of wild-type Huntingtin spanning from 1–171 to full length of 3144aa in three different cell types [47–49]. To observe the morphology phenotype in mammalian cells under conditions of Huntingtin reduction, it may also be necessary to use primary cells such as fibroblasts since transformed cells may have both altered PI 3-kinase signaling and changes in adhesion and motility that aid in metastasis. It is noteworthy that fifty percent reduction of Huntingtin protein levels in our cell cultures was sufficient to shift morphology profiles in the population.

Our data suggests that while normal Huntingtin is not necessary for the initial response to the growth factor PDGF (propagation of signal from the receptor tyrosine kinase and activation of PI 3-kinase to initiate actin based membrane ruffling and initial dissolution of adhesions), Huntingtin contributes to initiation, maturation or stabilizing or adhesion plaques, potentially by fostering local accumulations of α-actinin on membranes which promotes new focal contacts for adhesions. In contrast to the effects of Huntingtin lowering, the presence of the HD mutation in Huntingtin changed morphology in a small population of fibroblasts and suppressed the morphology response to growth factor stimulation. An increased number of mature Vinculin-positive focal adhesions occurred in HD fibroblasts compared to controls in normal growth medium. A suppressed response to signaling is consistent with previous results using microglia from HD patients compared to controls which showed reduced membrane ruffling in response to stimuli [50]. Whether this change is due to a suppression in upstream signaling, a resistance to dissolution of adhesion plaques, or increased maturation of focal adhesions is still to be determined.

Studies in Dictostelium and in mouse neural stem cells have implicated a role for normal Huntingtin in morphology changes and cell motility [51–53]. Studies in mice also indicate a role for Huntingtin in cell migration [9–11, 54–56]. In HD populations mammary tumors appear earlier and are more invasive and mutant Huntingtin increases cell motility supporting the notion that mutant Huntingtin affects adhesion and/or actin dynamics; furthermore, reduction of normal Huntingtin correlates with increased invasive carcinoma [57, 58]. Altered coordination of molecular complexes involved in adhesion and altered actin-based morphology caused by the absence of Huntingtin could account for these phenotypes.

Previous immunofluorescence work suggested co-localization of Huntingtin with actin. Miller at al. used monoclonal antibody 2166 and found labeling occurred in lamellipodia in mouse clonal striatal STHdh^Q111/Q111^ cells expressing mutant Huntingtin [20]. Hughes and colleagues also reported co-localization of an exogenously expressed Huntingtin fragment (1–558) fused to GFP with F-actin and the protein BAIAP2 in filipodia in mouse NIH3T3 cells [17]. Our data show more extensive localization of endogenous Huntingtin to the actin cytoskeleton in human fibroblasts (stress fibers, stellate polygonal structures, immature adhesion contacts, ruffled membranes and the leading edge of lamellipodia) than previously appreciated. Our anti-Huntingtin antibodies (Ab2527, Ab1173) are directed to more C-terminal domains in Huntingtin than those used before and this may have improved the extent of Huntingtin localization associated with the actin cytoskeleton. Huntingtin is a large protein (3144 aa) which is predicted to adopt numerous tertiary protein conformations when bound by interactors [59, 60] and antibodies directed to epitopes throughout the length of the Huntingtin protein can show quite different patterns of immunostaining in cells [40, 49, 61–64]. The epitopes for Ab1173 and Ab2527 also may be subject to posttranslational modifications which regulate intracellular localization to specific structures such as adhesion plaques and stress fibers. Both peptides used for immunizations to produce Ab 1173 and Ab2527 contain serines and at least one tyrosine. Both peptides also contain lysines which could be acetylated.

α-actinins are actin binding proteins that bundle and crosslink actin filaments in both contractile and non-contractile cells [34, 35]. α-actinin is recruited to focal complexes relatively early where it plays a structural role linking actin microfilaments to integrins and persists during hierarchical assembly as they mature to focal adhesions [28, 30, 32]. Previously published interactomes showed Huntingtin associated with α-actinin-1, -2 and -4 [15, 16], but these studies were not further validated. Our results from immunoprecipitations using exogenously expressed proteins, immunofluorescent co-localization and proximity ligation assay together with the altered localization of α-actinin with Huntingtin depletion in cells strongly support a functional interaction of Huntingtin with isoforms of α-actinin. Our results in primary fibroblasts and neurons along with work by others [3, 46, 65] suggest a model in which Huntingtin regulates α-actinin-1 localization and couples growth factor signaling with actin polymerization and with bundling functions at new sites of adhesion.

Where-as stimulation with PDGF caused increased levels of α-actinin staining on ruffles and its localization to focalized regions in cells treated with the control siRNA, PDGF stimulation caused decreased staining and a loss of focal staining in cells treated with Huntingtin siRNA. This finding supports the hypothesis that Huntingtin regulates stimulus dependent localization of α-actinin on the plasma membrane which may be necessary for focal contact restructuring following cytoskeletal reorganization. We speculate that the focal regions are sites of PI 3-kinase activity. Curiously, in serum starved conditions the levels of alpha-actinin were higher on membrane ruffles in cells with Huntingtin lowering compared to cells treated with a GFP siRNA. α-actinins bind to both PI(4,5)P2 and PI(3,4,5)P3; PI(3,4,5)P3 is produced by PI 3-kinase activity and is less abundant in the membrane compared to PI(4,5)P2 levels. Huntingtin also binds PI(4,5)P2 with a low affinity but binds the PI 3-kinase products PI(3,4)P2 and PI(3,4,5)P3 with higher affinity. Perhaps in the absence of Huntingtin, α-actinin binds to PI(4,5)P2 which is more abundant in the membrane, but the interaction of Huntingtin and α-actinin promotes high affinity binding to PI 3-kinase products at highly specialized regions. Our results suggest that Huntingtin is not essential for α-actinin to localize to membranes but that Huntingtin may be necessary for its redistribution to foci which may be destined to become new adhesion sites. The localization of Huntingtin to focal contacts and developing adhesions plaques together with its exclusion from mature adhesion plaques supports the notion the Huntingtin function is necessary early in plaque formation and that once the plaque is mature, Huntingtin is no longer needed.

α-actinin-2 is enriched in dendritic spines in brain [66], where it is highly dynamic [67] and regulates spine morphology and maturation [37] and the transport of the AMPA subtype of glutamate receptors to post-synaptic sites [68]. Conditional knockout of normal Huntingtin revealed that Huntingtin is necessary for development of excitatory synapses in the cortical-striatal pathways through an unknown mechanism [69]. Thus, Huntingtin-α-actinin interactions may be important for maturation and function of excitatory synapses.

The interaction between mutant Huntingtin and α-actinin isoforms could interfere with the function of dendritic spines in medium spiny neurons which are the most affected in HD patients. α-actinin is regulated by PI(3,4,5)P3 to which mutant Huntingtin binds much more avidly [33]. Thus, competition for PI(3,4,5)P3 may alter normal localization of α-actinin in HD cells. Studies suggest that there are lower levels of α-actinin in striatum of HD mouse models than in WT [70, 71]. Whether mutant Huntingtin interacts with α-actinin-2 at membranes of dendritic spines to affect its levels and localization remains to be determined.

The region in Huntingtin that interacts with α-actinin-2 is contained within aa 399–969. Additional amino acids N-terminal to Huntingtin residue 399 may be required for full interaction. Phosphorylation at serines 419 and 421 which are residues subject to phosphorylation by AKT a substrate of PI 3-kinase was not required. However, phosphorylation at one of these sites might regulate dissociation of or prevent the interaction between Huntingtin and α-actinin-2. This region in Huntingtin also contains the binding region for dynein [72]. A switch between microtubule-dependent and microfilament (actin-based) vesicle motility was previously described where Rab5-Hap40-Huntingtin bound more to microtubules [73]. We speculate that this switch to actin filaments may be mediated through competitive binding between α-actinins and dynein which both bind in the same region of Huntingtin.

In summary, our data support an important role for Huntingtin in cell morphology at the level of actin filaments, nascent focal adhesions, and membrane ruffles and suggest that Huntingtin is part of the fundamental machinery linking the actin cytoskeleton to membranes. Similarities between focal adhesion sites and synapses containing α-actinin have been recognized before [66]. The same molecular players that are responsible for morphology changes in fibroblasts, including α-actinin, are also important for regulating morphology of developing neurons and dendritic spines. Hence, the novel functions for Huntingtin described here may be important for the development and maintenance of normal spine morphology and explain the marked changes in dendritic spine morphology of medium spiny neurons reported in the brain of HD patients [74].

## Supporting information

S1 Fig Huntingtin depletion in human fibroblasts alters cell morphology but does not alter Caspase 3 processing. **(a)** Western blot analysis shows levels of Huntingtin protein are reduced in fibroblasts treated with siRNA E1-4 targeting Huntingtin compared to a control siRNA targeting GFP. Blots were probed with GAPDH as a loading control. Signal intensity results are shown in Fig 1a. (**b)** Western blots of lysates from mock transfected (transfection reagent alone), or transfected with siRNAs targeting GFP or Huntingtin (HTT) mRNA probed for Caspase 3 to look for evidence of apoptosis. All lysates showed similar amounts of unprocessed Caspase 3. Even long exposures (middle blot) show no evidence of 17kDa proteolytic fragment of Caspase3. (**c**) Control 1 fibroblast were transfected with Cy3 tagged siRNA targeting GFP mRNA to visualize proportion of transfected cells (top panel). Cell counts showed 79% ± 0.06 cells were positive for Cy3 (appeared as red punta concentrated within the cytoplasm of cells) compared to mock transfected cells with 0% (bottom panel (n = 3 coverslips). **(d)** Graph of data presented in Fig 1b expressed as a percentage of cells counted for each group and treatment.
(TIF)

S2 Fig Morphology changes in Control and HD cell lines in response to PDGF. **(a)** Graphs show mean percentage ±SD of Type 1 and Type2 cells in serum-starved Control 2 cells with or without 100 ng/ml PDGF for 15 minutes and counted as in Fig 1f. Control2 cells respond normally by inducing cells to ruffle as reflected by the reduction in Type1 cells and increase in Type2 cells (n = 6 coverslips, paired t-test, *<0.05. **(b)** Example of Type8 cell observed by Alexa-phalloidin staining and present HD2 cells. Type8 cells were long, spindly bipolar cells. **(c)** Graph shows mean ±SD of Type8 cells in Contorl1, HD1, and HD2 cultures. A significant increase in Type 8 cells in the HD 2 20/50 line did not change with PDGF treatment (Two-way ANOVA and Bonferroni posthoc test, *p<0.05, **p<0.01, n = 6 coverslips). Numbers in parentheses on x-axis indicate CAG repeat length for each Huntingtin allele in each cell. **(d)** Graph shows mean ±SD of Type8 cells in Control 2 cultures with and without 100 ng/ml PDGF for 15 minutes.
(TIF)

S3 Fig Huntingtin immunoreactivity using Ab2527 localizes to phalloidin-positive stress fibers in human fibroblasts and is resistant to treatment with brefeldin A. Control human fibroblasts were cultured for 48 hours on glass coverslips then treated with normal growth medium or medium containing 2 μg/ml brefeldin A (BFA) for 120 seconds. Cells were quickly washed with PBS, then fixed with PFA and stained for Huntingtin using Ab2527 (green) or F-actin with rhodamine-phalloidin (red). Sequential confocal images for each label were acquired. Co-localization is in merged image (yellow) at stress fibers (arrowheads). Huntingtin immunoreactivity on perinuclear vesicles (top panel, arrow) is lost with BFA treatment, but staining of stress fibers remains (bottom panel).
(TIF)

S4 Fig Full blots of SDS-PAGE and western blot analysis of immunoprecipitations performed for deletion analysis shown in Fig 5b and 5c. **(a)** *Top blot*, Western blots performed for data shown in Fig 5b probed first with an antibody targeting Huntingtin aa1-17 (Ab1). Endogenous full length Huntingtin and FLAG-HTT-1-3144 migrate at top of gel (Full length Htt, arrow, predicted molecular mass ~350 kDa). FLAG-HTT-1-969 migrated at ~135kDa (arrow). A product of ~72 kDa was also observed with overexpression of N-terminal HTT fragments. Ab1 cross-reacted with the heavy (56 kDa) and light chains (25 kDa) from the anti-FLAG M2 MAB antibody in IP lanes. Lanes containing FLAG-HTT-399-3144 were excluded from the main figure since expression of protein could not be demonstrated using an antibody to an epitope within HTT aa1173-1196 and may require antigen recapture (see also c and d). *Bottom blot*, Blot was re-probed with anti-α-actinin-2 antibody which recognized the exogenously expressed GFP-α-actinin-2 fusion protein (~130 kDa, arrow). **(b)** *Top blot*, Western blots using Ab1 of immunoprecipitations showing interactions with an N-terminal fragment containing a deletion of the proline rich region (FLAG-HTT-1-969 pro del) or containing site mutations S419G and S421G (FLAG-HTT-1-969 419GGG), a C-terminal fragment (FLAG-HTT-2569-3144), and an internal fragment (FLAG-HTT-399-1518). Ab1 recognizes the two N-terminal fragments (FLAG-HTT-1-969 pro del) (FLAG-HTT-1-969 419GGG (arrows) and endogenous full Huntingtin in input lysates (Full length Htt, arrow). *Bottom blot*, Blot was re-probed with anti-α-actinin-2 antibody which recognized the exogenously expressed GFP-alpha-actinin-2 fusion protein (~130 kDa, arrow). **(c)** Immunoprecipitations (same reactions analyzed in a and b) from full length and internal and C-terminal fragments of Huntingtin (FLAG-HTT-1-3144, FLAG-HTT-399-3144, and FLAG-HTT-2569-3144, and FLAG-HTT-399-1518) were run on a new blot and probed with Ab1173 targeting an epitope within HTT aa1173-1196 to confirm their expression. (These deletion constructs do not contain the epitope recognized by Ab1 used in a and b.) Blot confirmed expression of FLAG-HTT-1-3144 and FLAG-HTT-399-1518 (arrows). **(d)** Immunoprecipitation of FLAG-HTT-399-3144 using mAb 2166 and detected with anti-FLAG antibody M2 (arrow at about 300 kD, top blot) shows FLAG-HTT-399-3144 can interact with GFP-α-actinin-2 fusion protein (bottom blot). Detection of FLAG-HTT-399-3144 with M2 was only possible after stripping with a buffer at pH13.3 (see Methods) then neutralization with Tris buffer at pH 7.4 suggesting antigen recapture might be necessary to detect this fusion protein. The IPs were eluted into 75 μl of SDS-PAGE sample buffer containing DTT and boiled, then 15 μl/ well were loaded onto 3–8% Tris-acetate gels.
(TIF)

S5 Fig **(a) A proximity ligation assay (PLA) produced reaction product in ruffled membrane when an antibody for Huntingtin (Ab1173) and α-actinin-1 were used.** Fluorescence was visualized by confocal microscopy in human Control 1 fibroblasts (**a**). Left, the fluorescence confocal image shows the reaction product (Red) with anti-Huntingtin polyclonal Ab1173 and monoclonal anti- α-actinin-1 in small foci in the cytoplasm (arrows). Reaction product occurred rarely at ruffled membranes (large arrow) (Red, left column) were merged with the phase images taken sequentially (center column). The Merge image is shown in right column. Nucleus stained with Hoechst (blue). Fibroblasts grown with serum. Confocal images taken with 60X oil objective. **(b and c) Example of three regions of interest (ROIs) on the plasma membrane of a fibroblast cell.** (**b**) Cell stained for α-actinin (red) and Alexa-phalloidin (green). Three regions of interest per cell were selected at lamellapodia or areas of membrane ruffles viewed in Alexa-phalloidin channel. ROIs of 10 square pixels were placed so that the edge of the plasma membrane transited the center of the box leaving about half the box empty. Mean intensity per square pixel was determined and the average of three ROIs per cell calculated. 10 cells per coverslip were analyzed. Each coverslip was a biological replicate (individual siRNA treatment after plating), n = 3 coverslips per condition. N = nucleus. (**c**) Sample images show α-actinin localization uniformly distributed at the plasma membrane in fibroblast cells treated with Huntingtin siRNA E1-4 showing Type 6 morphology. Scale bar = 50 μm.
(TIF)

## Abbreviations

BFA: brefeldin A
HD: Huntington Disease
Htt: Huntingtin
IPSC: induced pluripotent stem cell
MS: mass spectrometry
NSC: neural stem cell
PDGF: platelet derived growth factor
PI 3: kinase, phosphatidylinositol 3-OH kinase
PI(3,4)P2: phosphatidylinositol 3,4-bisphosphate
PI(3,4,5)P3: phosphatidylinositol 3,4,5-triphosphatepolyQ, polyglutamine
WT: wild-type
