# Supplementary figures and images for: Huntingtin associates with the actin cytoskeleton and α-actinin isoforms to influence stimulus dependent morphology changes
Source: PLoS One. 2019 Feb 15;14(2):e0212337. doi: 10.1371/journal.pone.0212337 (PMC6377189; doi:10.1371/journal.pone.0212337)

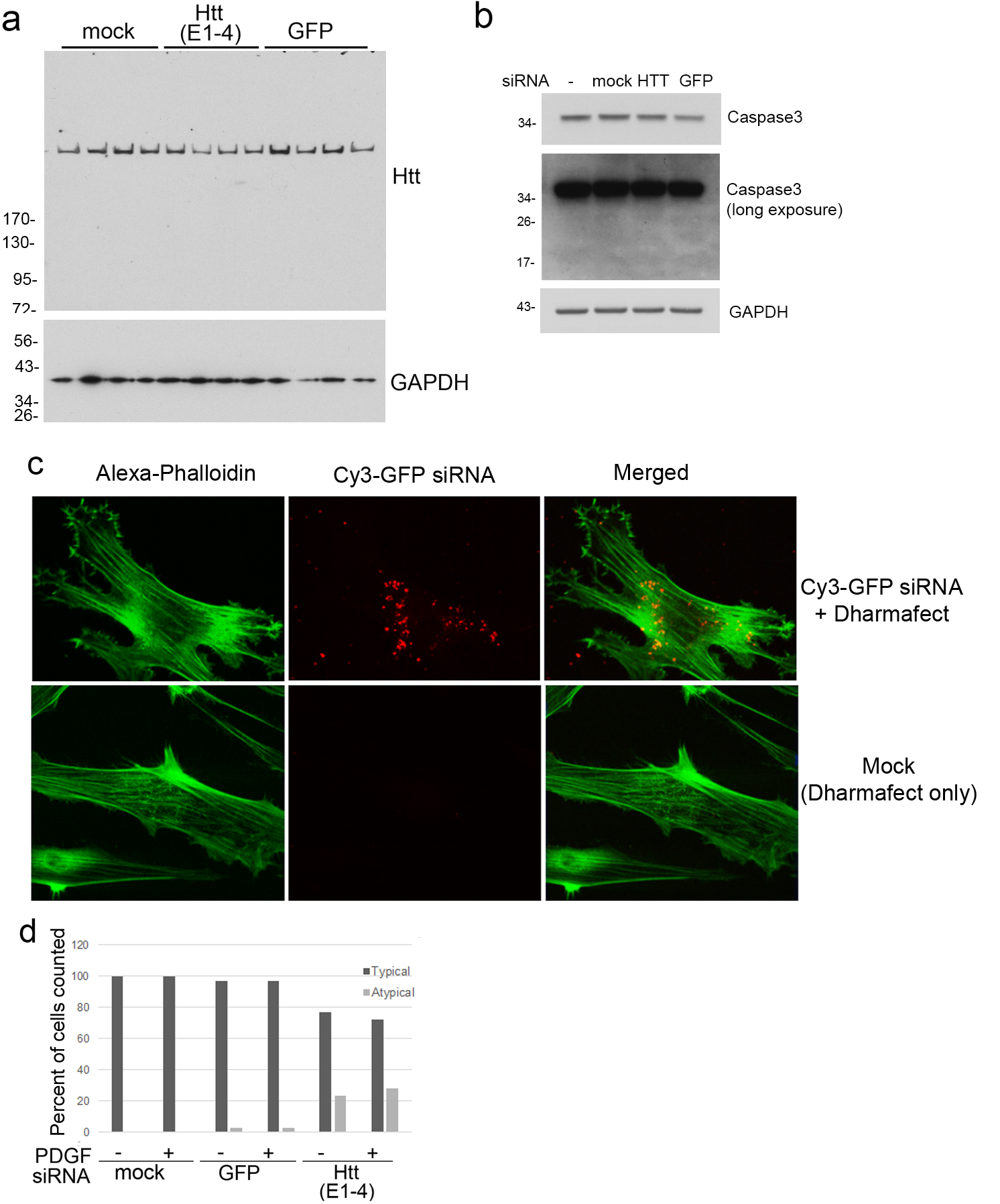

Supplement: S1 Fig — (a) Western blot analysis shows levels of Huntingtin protein are reduced in fibroblasts treated with siRNA E1-4 targeting Huntingtin compared to a control siRNA targeting GFP. Blots were probed with GAPDH as a loading control. Signal intensity results are shown in Fig 1a. (b) Western blots of lysates from mock transfected (transfection reagent alone), or transfected with siRNAs targeting GFP or Huntingtin (HTT) mRNA probed for Caspase 3 to look for evidence of apoptosis. All lysates showed similar amounts of unprocessed Caspase 3. Even long exposures (middle blot) show no evidence of 17kDa proteolytic fragment of Caspase3. (c) Control 1 fibroblast were transfected with Cy3 tagged siRNA targeting GFP mRNA to visualize proportion of transfected cells (top panel). Cell counts showed 79% ± 0.06 cells were positive for Cy3 (appeared as red punta concentrated within the cytoplasm of cells) compared to mock transfected cells with 0% (bottom panel (n = 3 coverslips). (d) Graph of data presented in Fig 1b expressed as a percentage of cells counted for each group and treatment. (TIF) [file pone.0212337.s001.tif]

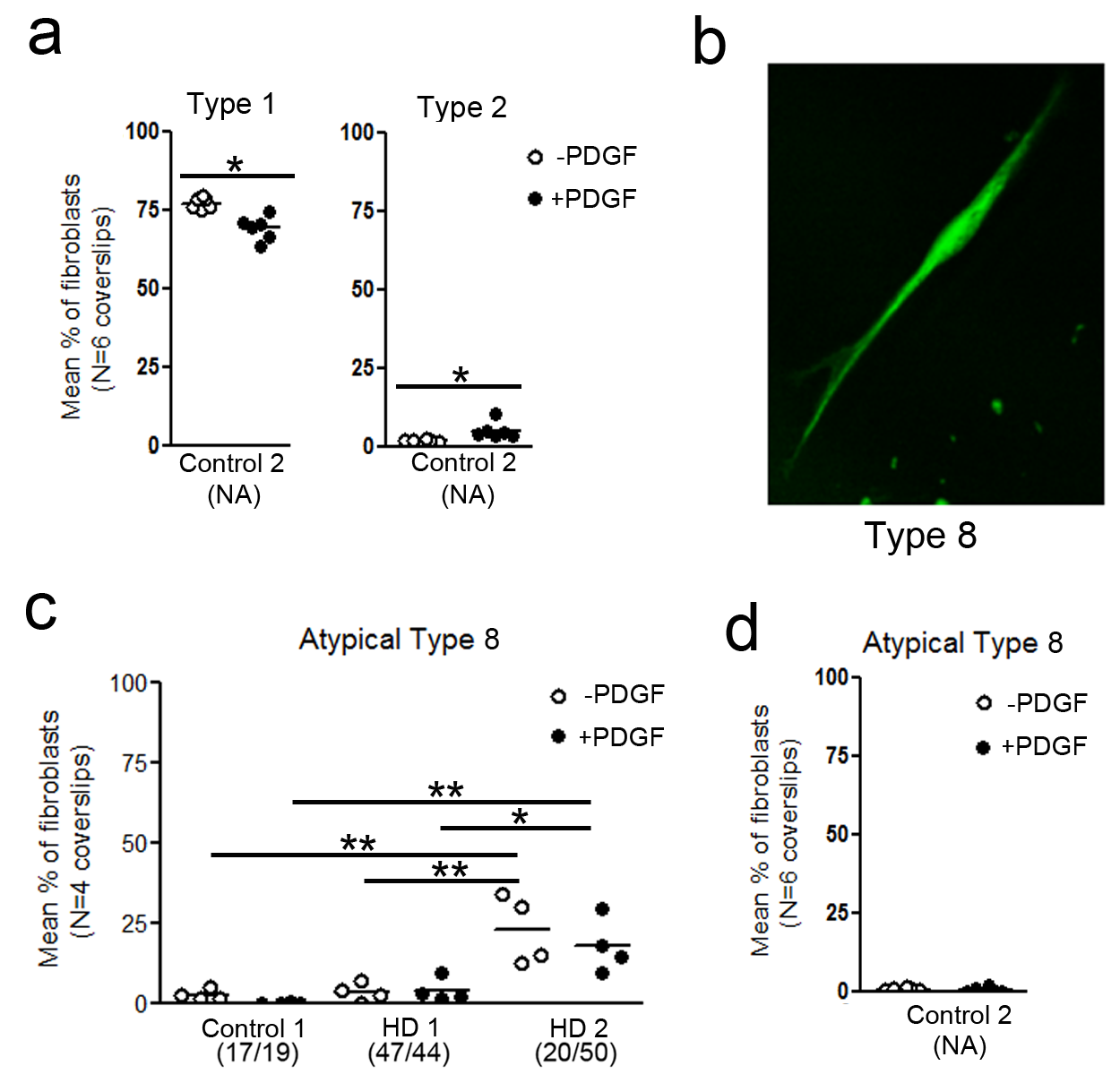

Supplement: S2 Fig — (a) Graphs show mean percentage ±SD of Type 1 and Type2 cells in serum-starved Control 2 cells with or without 100 ng/ml PDGF for 15 minutes and counted as in Fig 1f. Control2 cells respond normally by inducing cells to ruffle as reflected by the reduction in Type1 cells and increase in Type2 cells (n = 6 coverslips, paired t-test, *<0.05. (b) Example of Type8 cell observed by Alexa-phalloidin staining and present HD2 cells. Type8 cells were long, spindly bipolar cells. (c) Graph shows mean ±SD of Type8 cells in Contorl1, HD1, and HD2 cultures. A significant increase in Type 8 cells in the HD 2 20/50 line did not change with PDGF treatment (Two-way ANOVA and Bonferroni posthoc test, *p<0.05, **p<0.01, n = 6 coverslips). Numbers in parentheses on x-axis indicate CAG repeat length for each Huntingtin allele in each cell. (d) Graph shows mean ±SD of Type8 cells in Control 2 cultures with and without 100 ng/ml PDGF for 15 minutes. (TIF) [file pone.0212337.s002.tif]

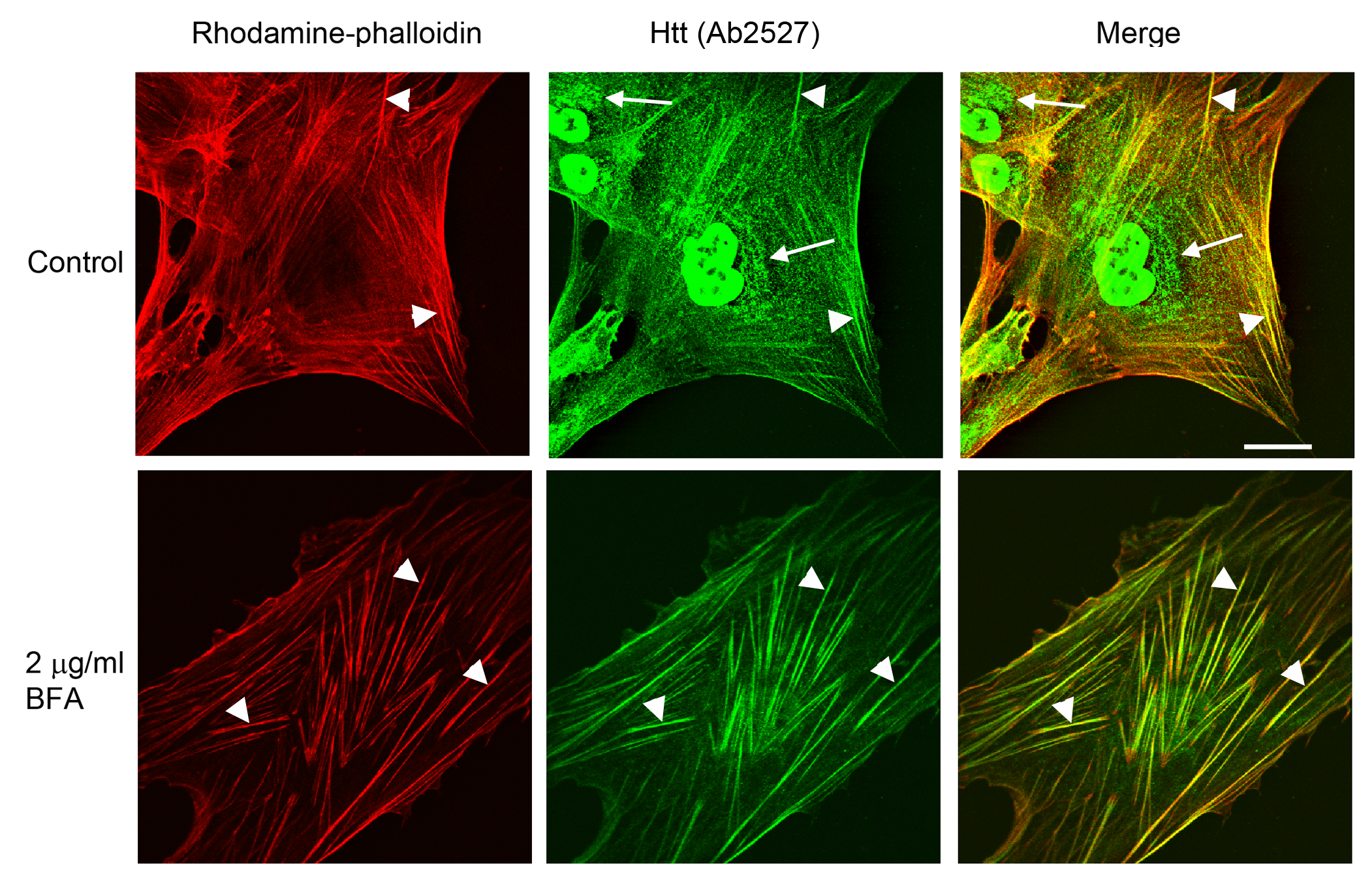

Supplement: S3 Fig — Control human fibroblasts were cultured for 48 hours on glass coverslips then treated with normal growth medium or medium containing 2 μg/ml brefeldin A (BFA) for 120 seconds. Cells were quickly washed with PBS, then fixed with PFA and stained for Huntingtin using Ab2527 (green) or F-actin with rhodamine-phalloidin (red). Sequential confocal images for each label were acquired. Co-localization is in merged image (yellow) at stress fibers (arrowheads). Huntingtin immunoreactivity on perinuclear vesicles (top panel, arrow) is lost with BFA treatment, but staining of stress fibers remains (bottom panel). (TIF) [file pone.0212337.s003.tif]

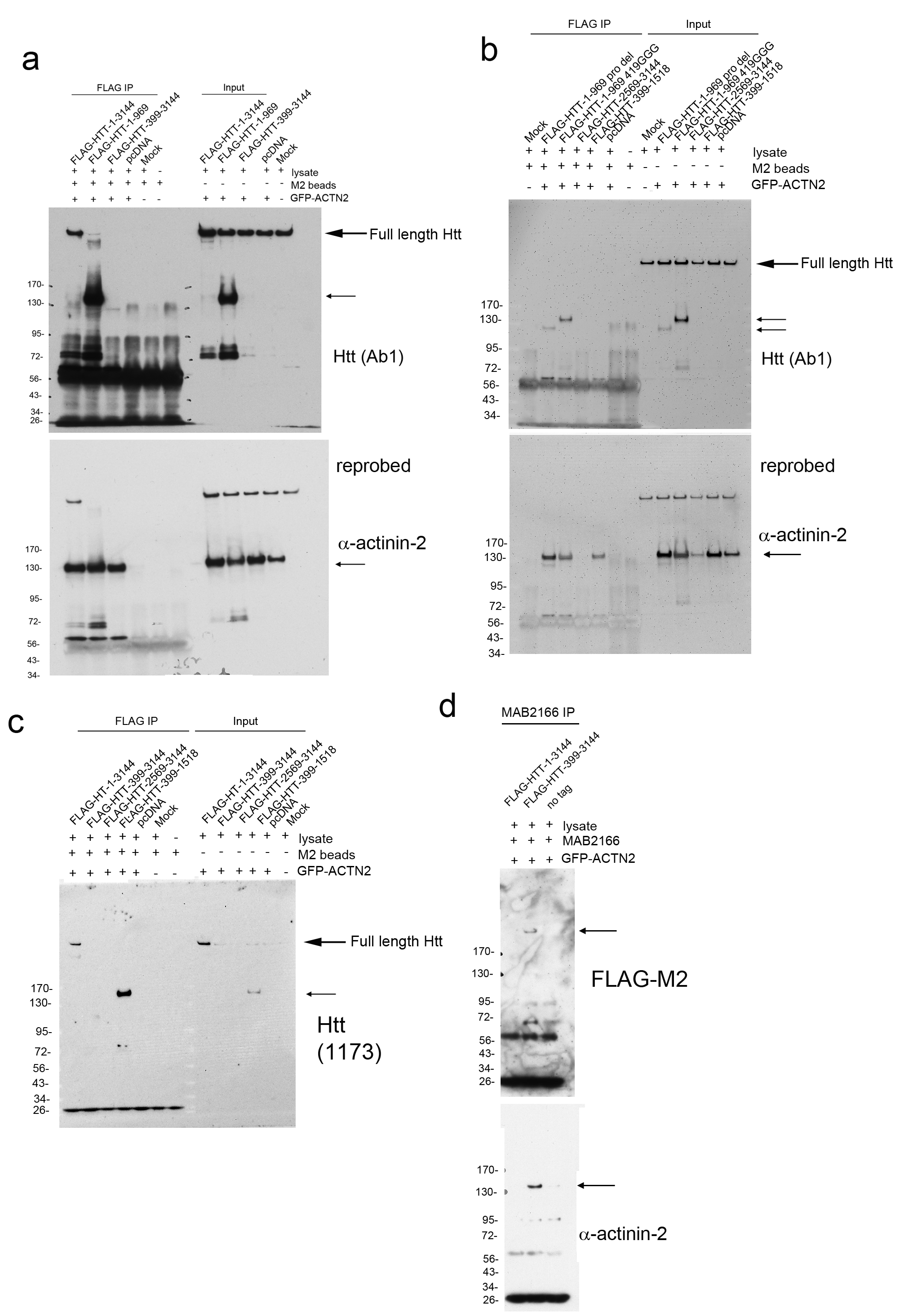

Supplement: S4 Fig — (a) Top blot, Western blots performed for data shown in Fig 5b probed first with an antibody targeting Huntingtin aa1-17 (Ab1). Endogenous full length Huntingtin and FLAG-HTT-1-3144 migrate at top of gel (Full length Htt, arrow, predicted molecular mass ~350 kDa). FLAG-HTT-1-969 migrated at ~135kDa (arrow). A product of ~72 kDa was also observed with overexpression of N-terminal HTT fragments. Ab1 cross-reacted with the heavy (56 kDa) and light chains (25 kDa) from the anti-FLAG M2 MAB antibody in IP lanes. Lanes containing FLAG-HTT-399-3144 were excluded from the main figure since expression of protein could not be demonstrated using an antibody to an epitope within HTT aa1173-1196 and may require antigen recapture (see also c and d). Bottom blot, Blot was re-probed with anti-α-actinin-2 antibody which recognized the exogenously expressed GFP-α-actinin-2 fusion protein (~130 kDa, arrow). (b) Top blot, Western blots using Ab1 of immunoprecipitations showing interactions with an N-terminal fragment containing a deletion of the proline rich region (FLAG-HTT-1-969 pro del) or containing site mutations S419G and S421G (FLAG-HTT-1-969 419GGG), a C-terminal fragment (FLAG-HTT-2569-3144), and an internal fragment (FLAG-HTT-399-1518). Ab1 recognizes the two N-terminal fragments (FLAG-HTT-1-969 pro del) (FLAG-HTT-1-969 419GGG (arrows) and endogenous full Huntingtin in input lysates (Full length Htt, arrow). Bottom blot, Blot was re-probed with anti-α-actinin-2 antibody which recognized the exogenously expressed GFP-alpha-actinin-2 fusion protein (~130 kDa, arrow). (c) Immunoprecipitations (same reactions analyzed in a and b) from full length and internal and C-terminal fragments of Huntingtin (FLAG-HTT-1-3144, FLAG-HTT-399-3144, and FLAG-HTT-2569-3144, and FLAG-HTT-399-1518) were run on a new blot and probed with Ab1173 targeting an epitope within HTT aa1173-1196 to confirm their expression. (These deletion constructs do not contain the epitope recognized by Ab1 used in a an [file pone.0212337.s004.tif]

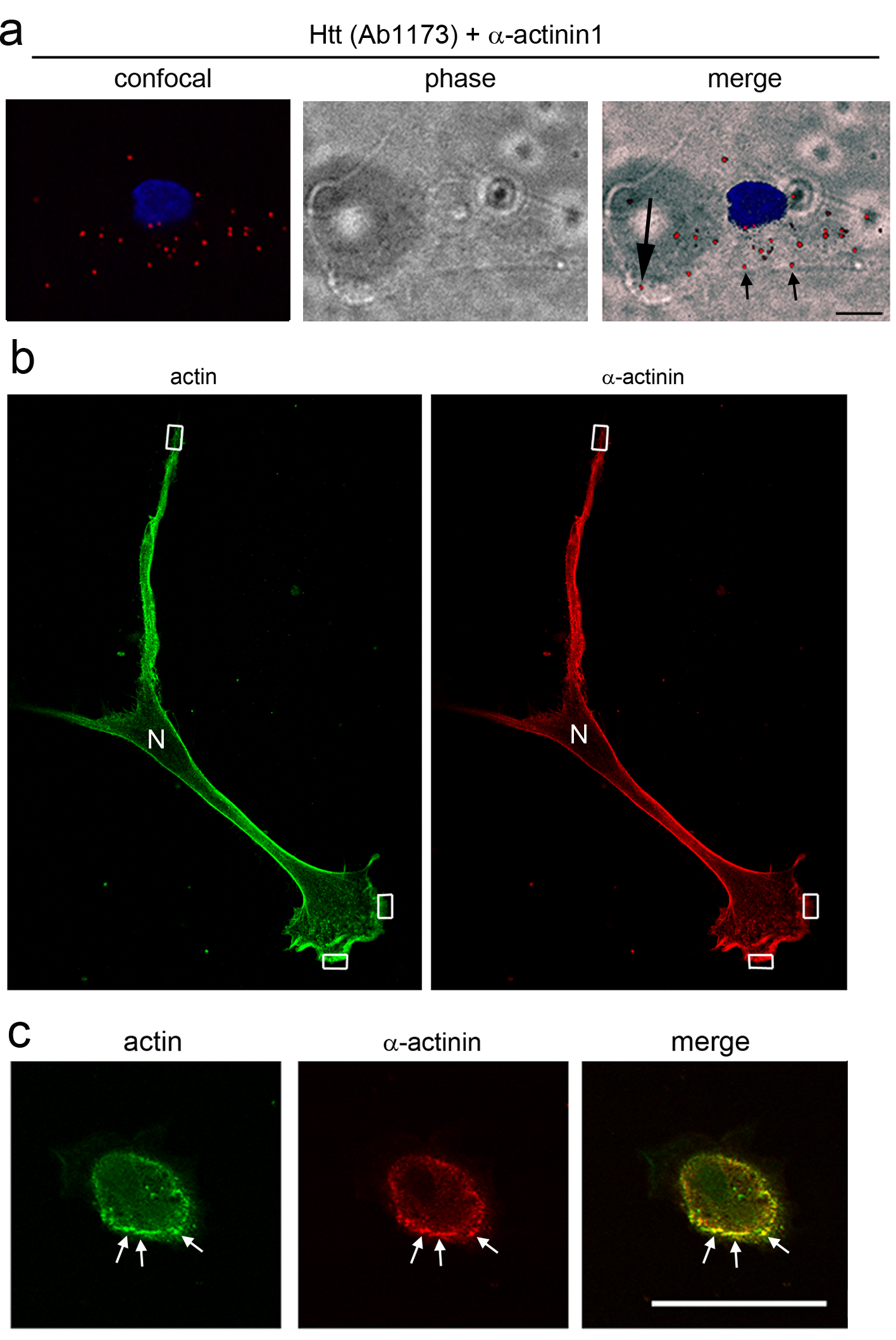

Supplement: S5 Fig — (a) A proximity ligation assay (PLA) produced reaction product in ruffled membrane when an antibody for Huntingtin (Ab1173) and α-actinin-1 were used. Fluorescence was visualized by confocal microscopy in human Control 1 fibroblasts (a). Left, the fluorescence confocal image shows the reaction product (Red) with anti-Huntingtin polyclonal Ab1173 and monoclonal anti- α-actinin-1 in small foci in the cytoplasm (arrows). Reaction product occurred rarely at ruffled membranes (large arrow) (Red, left column) were merged with the phase images taken sequentially (center column). The Merge image is shown in right column. Nucleus stained with Hoechst (blue). Fibroblasts grown with serum. Confocal images taken with 60X oil objective. (b and c) Example of three regions of interest (ROIs) on the plasma membrane of a fibroblast cell. (b) Cell stained for α-actinin (red) and Alexa-phalloidin (green). Three regions of interest per cell were selected at lamellapodia or areas of membrane ruffles viewed in Alexa-phalloidin channel. ROIs of 10 square pixels were placed so that the edge of the plasma membrane transited the center of the box leaving about half the box empty. Mean intensity per square pixel was determined and the average of three ROIs per cell calculated. 10 cells per coverslip were analyzed. Each coverslip was a biological replicate (individual siRNA treatment after plating), n = 3 coverslips per condition. N = nucleus. (c) Sample images show α-actinin localization uniformly distributed at the plasma membrane in fibroblast cells treated with Huntingtin siRNA E1-4 showing Type 6 morphology. Scale bar = 50 μm. (TIF) [file pone.0212337.s005.tif]
